# Chemical characteristics, antioxidant capacity, bacterial community, and metabolite composition of mulberry silage ensiling with lactic acid bacteria

**DOI:** 10.3389/fmicb.2024.1363256

**Published:** 2024-04-08

**Authors:** Yaya Guo, Rongzheng Huang, Yujie Niu, Peng Zhang, Yuan Li, Wenju Zhang

**Affiliations:** College of Animal Science and Technology, Shihezi University, Shihezi, China

**Keywords:** mulberry silage, epiphytic lactic acid bacteria, antioxidant capacity, bacterial community, metabolites composition

## Abstract

Mulberry has high crude protein and biologically active compounds but is difficult to be ensiled due to the lack of adequate epiphytic LAB. This study aimed to investigate the effects of inoculation with *Lactiplantibacillus plantarum* and *Pediococcus pentosaceus* isolated from mulberry with higher antioxidant capacity alone or in combination with *Streptococcus bovis* on chemical characteristics, antioxidant capacity, bacterial community, and metabolite composition of mulberry silage. The results showed that all inoculation groups had higher dry matter and lower pH than the control group, particularly in LP (dry matter, DM, 32.03% and pH **=** 4.44) and LP_PP_SB (DM, 31.68% and pH = 4.26) after 60 days of ensiling. Ammonia nitrogen (AN) content was the lowest in both LP_SB and LP_PP_SB groups, which were 1.86 g/kg FM and 1.05 g/kg FM, respectively, (*P* < 0.05). Only the LP_PP_SB group showed increased polyunsaturated fatty acids (PUFA, 1.2851 g/kg DM, *P* < 0.05) than the control group. Ferric-reducing antioxidant power (FRAP) values were increased in all inoculation-treated groups compared with the control group (*P* < 0.05). 2,2-Diphenyl-1-picrylhydrazyl (DDPH), 3-ethylbenzothiazoline-6-sulfonic acid (ABTS), and FRAP exhibited the highest levels in the LP_PP- and LP_PP_SB-treated groups. *Enterobacter* was dominant in both the control and SB-treated groups, and the relative abundance was 41.18% and 32.35%, respectively (*P* < 0.05). The relative abundance of *Lactiplantibacillus* was higher in the LP-, LP_PP-, and LP_SB-treated groups (81.84%−82.69%). Relative abundance of *Pediococcus* was higher in the PP-, PP_SB-, and LP_PP_SB-treated groups (74.27%−85.27%). Untargeted metabolomics analysis results showed that five flavonoids (apigenin, eriodictyol, quercetin-3-glucoside, rutin, and kaempferol-3-O-rutinoside)were upregulated in all inoculation groups (except for the SB-treated groups). Among them, eriodictyol was both positively correlated with ABTS and FRAP and also showed the highest relative abundance in the LP_PP- and LP_PP_SB-treated groups. To the best of our knowledge, this study was the first to investigate the relationship between inoculants of epiphytic lactic acid bacteria and antioxidant capacity by 16s rRNA Illumina sequencing technology and untargeted metabolomics analysis, respectively. Consequently, inoculated *L. plantarum, P. pentosaceus* alone, respectively, or in combination with *S. bovis* increased the relative abundance of *Lactiplantibacillus* and *Pediococcus* and decreased the relative abundance of *Enterobacter*, particularly in the LP_PP_SB-treated group. In addition, inoculants could increase the relative abundance of five flavonoids (apigenin, eriodictyol, quercetin-3-glucoside, rutin, and kaempferol-3-O-rutinoside), especially eriodictyol to improve the antioxidant capacity of mulberry silage.

## 1 Introduction

Mulberry, which is widely distributed in the south of China, has been considered a new fodder resource that has replaced traditional fodder for ruminants for alleviating feed scarcity, as it possesses high crude protein content similar to that of alfalfa hay (20%, dry matter, DM) (Simbaya et al., 2020) and is rich in various bioactive compounds, such as quercetin (1.075–2.645 mg/g DM), rutin (0.976–2.714 mg/g DM), isoquercetin (0.101–0.870 mg/g DM), and kaempferol glycosides (0.281–0.615 mg/g DM) (Yu et al., 2019). These bioactive compounds possess biological functions that improve human and animal health, such as antitumor, anticancer, antioxidant, hypoglycemic, and lipid-lowering effects (Byong Tae, 2012; He et al., 2018). Among them, the antioxidant capacity of mulberry has received significant attention. The antioxidant capacity of silage is different in various cultivars of mulberry, DDPH (6.79–210.27 mg Trolox/100 g FW), FRAP (0.26–4.54 Fe mmol /100 g FW), and ABTS (mg ascorbic acid /100g FW) (Chen et al., 2016). Antioxidant capacity is crucial for health and production performance in ruminants, as oxidative stress is associated with metabolic diseases in ruminants (Hassan et al., 2020). Elevated levels of oxygen in ruminants could result in the formation of reactive oxygen species (ROS), including superoxide anion, hydrogen peroxide, and reactive hydroxyl radicals (Feng and Wang, 2020). Excessive accumulation of ROS may lead to oxidative stress or cell death by damaging proteins, DNA, and lipids (Feng and Wang, 2020), triggering various metabolic diseases such as sepsis, acidosis, mastitis, and ketosis, thereby affecting the health and production performance in ruminants (Piao et al., 2023). Fresh mulberry was subject to seasonal limitations in ruminants, and hence drying or ensiling of mulberry was needed for it to be preserved for a long time. However, haymaking presents some potential limitations, such as loss of both leaves and nutrients during production and shipping, and its storage process is subject to changes in weather. Therefore, silage has become increasingly popular due to its similarity in nutrient profile to fresh mulberry (Garcez Neto et al., 2018). In addition, various polyunsaturated fatty acids (PUFAs) present in forage can be transferred to milk or meat products of ruminant animals, while bioactive compounds may enhance the levels of PUFAs in dairy products (Ali et al., 2020). However, the compositions of fatty acids in mulberry have not been reported.

The successful silage fermentation process was due to the abundant presence of lactic acid bacteria (LAB), which produced a large amount of lactic acid through the fermentation of carbohydrates, and a decrease in the pH value inhibited microorganism activity under an anaerobic environment and enabled the preservation of forage for a long time (Rooke and Hatfield, 2015). However, the lack of adequate epiphytic LAB is one of the reasons for the difficult ensiling of mulberry (He et al., 2020); inoculated commercial or isolated LAB could solve this problem (Pitiwittayakul et al., 2021). Usually, epiphytic LABs are more effective than commercial inoculants due to the efficiency of carbohydrate utilization and adaptation to the environment (Chen et al., 2023). In addition, many LABs (such as *Lactobacillus helveticus* Li et al., 2019, *Lactobacillus sanfranciscensis*, and *Lactobacillus farciminis* Galli et al., 2018) exhibited excellent antioxidant capacity, which enables the production of antioxidant enzymes, such as superoxide dismutase (SOD) and catalase (CAT), to neutralize or alleviate reactive oxygen species (Vougiouklaki et al., 2023). Moreover, LAB inoculants would change the metabolic composition of mulberry silage, including flavonoids, by having an effect on the relative abundance and activity of microbial bacteria (Xia et al., 2022b). In addition, the abundance of bioactive compounds (such as phenolics and flavonoids) related to antioxidant capacity increased after fermentation (Kim and Jang, 2011). Therefore, inoculants of LABs with antioxidant capacity may increase the antioxidant capacity of mulberry silage by having an effect on metabolite composition. *S. bovis* could be an ideal starter inoculation to improve the efficacy of other LABs in fermentation (Jones et al., 1991). Therefore, the combination of epiphytic LAB inoculants with *S. bovis* would improve mulberry silage quality and antioxidant capacity by affecting the bacterial community, hence producing different metabolites such as flavonoids (He et al., 2021; Xia et al., 2022b). The food fermentation system inoculated with LAB could increase bioactive compounds (such as phenolics and flavonoids) and antioxidant capacity (Kim and Jang, 2011; Xia et al., 2022a). However, the mechanism of how epiphytic LAB affected silage quality and antioxidant capacity by altering the microbial community and metabolite composition is still unclear. Untargeted metabolomics analysis could be an ideal method for the comprehensive detection of qualitative and quantitative analysis of metabolites in the mulberry silage system (Fu et al., 2023).

Therefore, the main objective of this study was to investigate the impact of two LAB strains, isolated from naturally fermented mulberry silage with higher antioxidant capacity, when inoculated alone or in combination with *S. bovis*, on the chemical characteristics and antioxidant capacity of mulberry silage. In addition, we also used 16S rRNA Illumina sequencing technology and untargeted metabolomics analysis to elucidate how inoculation of epiphytic lactic acid bacteria affects the fermentation quality and antioxidant capacity of mulberry silage by altering the bacterial community and metabolites of silage.

## 2 Materials and methods

### 2.1 Strains source

Eight strains of *Leuconostoc mesenteroides* (LM14, ON937314; LM15, ON937315; LM17, ON937317; LM20, ON937320; LM21, ON937321; LM22, ON937322; LM23, ON937323; and LM27, ON937327), two strains of *L. plantarum* (LP19, ON937319 and LP26, ON937326), and two strains of *P. pentosaceus* (PP18, ON937318 and PP25, ON937325) were isolated from natural fermented mulberry silage. One of the *S. bovis* (SB87, OQ812187) strains was isolated from cattle rumen. Absorbance at 600 nm and the pH value of 12 LAB strains isolated from naturally fermented mulberry are shown in Supplementary Table S1. The methods for strain isolation and identification were adapted from Peng et al. (2021), and the phylogenetic tree, constructed based on the analysis of 16S rDNA sequences, is presented in Supplementary Figure S1.

### 2.2 Antioxidant capacity of LAB strains

The antioxidant capacity of LAB strains was determined following a previous method (Madjirebaye et al., 2022; Wang and Li, 2022), with slight modification. Specifically, we inoculated 3% strain in liquid de man rogosa and Sharpe (MRS) medium, centrifuged at 1800 r/min, incubated at 37°C for 18 h, and then centrifuged at 4°C at a speed of 3600×g, for 10 min, and then the liquid supernatant was extracted by filtering through a 0.22-μm membrane for preparing the cell-free supernatant. The antioxidant capacity of strains was determined via DDPH, ABTS, and FRAP methods, and operating procedures were carried out according to the manufacturer's kit (Suzhou Grace Biotechnology Co., Ltd., Suzhou, Jiangsu, China).

### 2.3 Materials and silage preparation

Mulberry was harvested in July at 10–15 cm height above the ground in Ili Kazakh Autonomous Prefecture, Xinjiang Province, China (80°09′42″ ~ 91°01′45″N, 40°14′16″ ~ 49°10′45″E). Whole mulberry plants were cut into 2 cm pieces using a grass fodder machine, and 1 kg of the sample was placed in polyethylene plastic bags (35 × 45 cm). LP26 and PP18 with higher antioxidant capacity, isolated from naturally fermented mulberry by conducting the assay given in Section 2.2, were inoculated alone or in combination with SB87 for ensiling mulberry: (1) CON: untreated; (2) SB: inoculated with SB87; (3) LP: inoculated with LP26; (4) PP: inoculated with PP18; (5) LP_PP: inoculated with LP26 and combined with PP18, inoculation ratio of 1:1; (6) LP_SB: inoculated with LP26 and combined with SB87, at an inoculation ratio of 1:1; (7) PP_SB: inoculated with PP18 and combined with SB87, at an inoculation ratio of 1:1; and (8) LP_PP_SB: inoculated with LP26 and combined with PP18 and SB87, at an inoculation ratio of 1:2:1. In each treated group, the inoculants were sprayed on mulberry at a concentration of 1 × 10^6^ colony-forming units (CFU)/g fresh weight (FW), and the same volume of distilled water was added to the control group. In total, 28 polyethylene silage bags (7 treatments × 4 replicates, ~1 kg/bag) were stored at 25°C, and samples were taken after 60 days to determine the chemical characteristics, antioxidant capacity, fatty acid, microbial community, and metabolite compositions.

### 2.4 Chemical characteristics, microbial community, antioxidant capacity, and fatty acid analysis

DM of fresh and silage mulberry was performed at 65°C to a constant weight. Contents of crude protein (CP), neutral detergent fiber (NDF), acid detergent fiber (ADF), and WSC concentration were determined according to previous methods (Arthur Thomas, 1977; Van Soest et al., 1991). The mulberry silage extract was filtered through four layers of gauze to determine the pH, AN, organic acids, and antioxidant capacity. Silage pH was determined using a pH meter (PHS-3C, Instrument and Electrical Science Instrument Co., Ltd., Shanghai, China). The content of AN was determined using anthrone colorimetry (Weatherburn, 1967). The concentration of organic acids was determined using high-performance liquid chromatography (HPLC) (Cui et al., 2022). The antioxidant capacity of the ferric-reducing antioxidant power (FRAP), 3-ethylbenzothiazoline-6-sulfonic acid (ABTS), and 2,2-diphenyl-1-picrylhydrazyl (DPPH) was measured using commercial kits (Suzhou Grace Biotechnology Co., Ltd). The fatty acid composition was qualitative and quantified according to the methods in the previous study (Sukhija and Palmquist, 1988). In total, 20 g of fresh and silage was mixed in 180 ml of sterile saline (0.9% sodium chloride) filtered through four layers of gauze and serially diluted to count LAB, yeast, and coliforms using the traditional plate count method. LAB counts were conducted on de man rogosa and Sharpe (MRS) agar at 37°C for 2 days. Yeast counts were performed on yeast extract peptone dextrose (YPD) agar at 30°C over a period of 2–3 days. Coliform counts were determined on Violet Red Bile (VRB) agar at 30°C for 2 days (Wang et al., 2018). All of the microbial culture media was provided by Qingdao Hi-tech Industrial Park Hope Biotechnology Co., Ltd.

### 2.5 Bacterial community of mulberry silage

Mulberry silage DNA was extracted using a bacterial DNA kit [Yeasen Biotechnology (Shanghai) Co., Ltd.], according to the manufacturer's instructions. The 16S rDNA V3–V4 regions were amplified using the upstream primer 338F (5′-ACTCCTACGGGAGGCAGCAG-3′) and the downstream primer 806R (5′-GGACTACHVGGGTWTCTAAT-3′) with the PCR conditions set as described by a previous study (Zhang et al., 2022). Amplicons were extracted, purified, and analyzed as previously described (Tahir et al., 2023). The PCR products were sequenced using the Illumina MiSeq PE250. The quality filtering, clustering, and analysis of 16S rRNA were performed using the Novogene Magic Platform and analyzed data as previously described (Tahir et al., 2023).

### 2.6 Mulberry silage metabolomics analysis

#### 2.6.1 Preparation of samples for untargeted metabolomics analysis

Frozen centrifuge tubes containing 50 mg of each sample of 60-day fermented mulberry were prepared. One grinding bead and 400 μl of 80% methanol were added to each tube to extract metabolites. The extraction solution was ground in a cryogenic grinder at −10°C and 50 Hz for 6 min. Then, it was extracted in a low-temperature ultrasonic extractor at 5°C and 40 kHz for 30 min and frozen in a −20°C freezer for another 30 min. Later, it was centrifuged at 4°C with a speed of 13000×g using a high-speed freezing centrifuge machine (Centrifuge5340, Eppendorf, Germany) for 15 min. The supernatant was used for analysis on the ultra-high-performance liquid chromatography (UHPLC)-Q EXACTIVE HF-X system.

#### 2.6.2 UHPL conditions

The HSS T3 chromatographic column (100 mm×2.1 mm i.d., 1.8 ml) was used with an injection volume of 3 μl, a flow rate of 0.40 ml/min, and a column temperature of 40°C for gradient elution separation. The mobile phase A consisted of 95% water: 5% acetonitrile (v/v, containing 0.1% formic acid), while the mobile phase B consisted of 47.5% acetonitrile:47.5% isopropanol:5% water (v/v/v, containing 0.1% formic acid). For positive ion mode elution gradient, the following conditions were applied: 0–3 min, 0%−20% B; 3–4.5 min, 20%−35% B; 4.5–5 min, 35%−100% B; 5–6.3 min, 100% B; at 6 0.3 min, 100%−0% B; 6.4–8 min, 0% B. For negative ion mode elution gradient, the following conditions were applied: 0–1.5 min, 0%−5% B; 1.5–2 min, 5%−10% B; 2.5–4.5 min, 10%−30% B; 4.5–5 min, 30%−100% B; 5–6.3 min, 100% B; 6.3–6.4 min, 100%−0% B, and 6.4–8 min, 0% B.

#### 2.6.3 Mass spectrometry analysis conditions

Mass spectrometry signals were acquired using both positive and negative ion scanning modes, covering a mass range of 70–1050 m/z. The sheath gas flow rate was set at 50 psi, while the auxiliary gas flow rate was maintained at 13 psi. Additionally, the temperature was controlled at 425°C. For the positive ion mode, the spray voltage was adjusted to −3500V, and the ion transfer tube temperature was set to 325°C. To ensure normalization, collision energy ranging from 40 to 60V in a cyclic manner was employed. The mass spectrometer operated at a resolution of 60,000, while the second-stage resolution remained fixed at 7,500. Data collection will be conducted using DDA mode.

### 2.7 Data analysis

Data of chemical characteristics, microbial counts, antioxidant capacity, and fatty acid composition were analyzed using a one-way ANOVA using the Statistical Package for the Social Sciences Statistics 26.0 (IBM Corp., Armonk, NY, United States). Differences among treated groups were analyzed by Tukey's test, and a *p* < 0.05 was considered a significant difference. All microbial data were analyzed using the Majorbio Cloud Platform (https://www.majorbio.com/web/www/index), and the bacterial sequences (SAR number for bacteria: PRJNA949551) were uploaded to the National Center for Biotechnology Information. Metabolites were analyzed using partial least squares discriminant analysis (PLS_DA) in the Majorbio Cloud Platform (https://www.majorbio.com/web/www/index). All metabolites were compared and identified on the Kyoto Encyclopedia of Genes and Genomes (KEGG) and Human Metabolome Database (HMDB). Differential metabolite analysis was carried out using Student's *t*-test with a significance level of *P* <0.05. Fold changes <1 were considered significant.

## 3 Results

### 3.1 Antioxidant capacity of LAB strains

**Figure 1:**
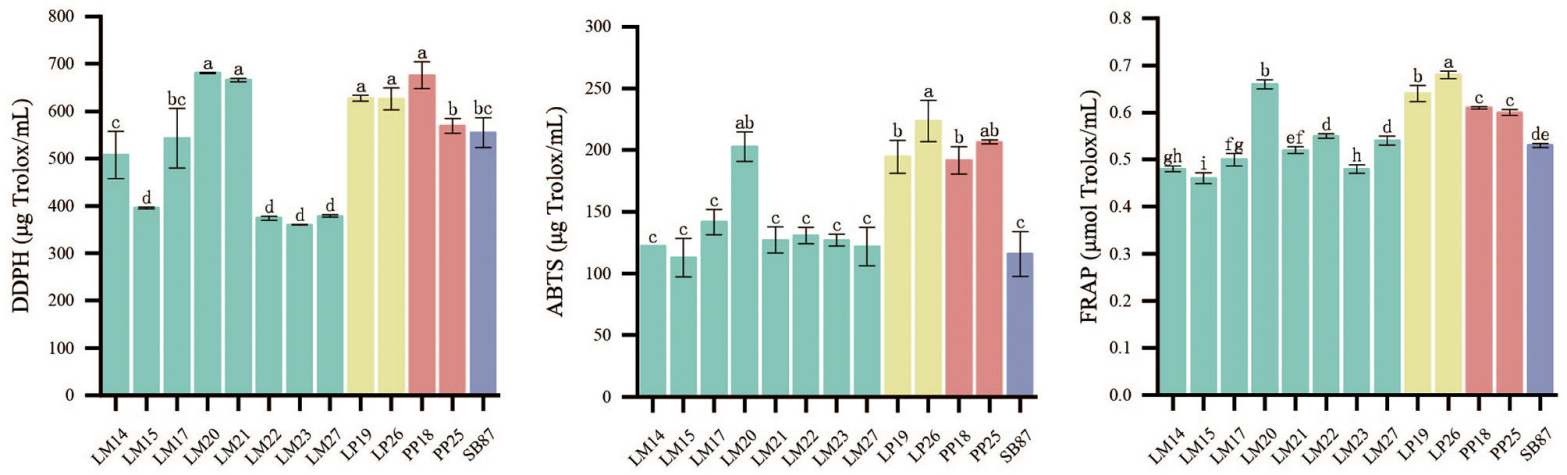
Antioxidant capacity of 13 LAB strains. LM, *L. mesenteroides;* LP, *L. plantarum;* PP, *P. pentosaceus;* and SB, *S. bovis*. Different lowercase letters on the bar represent significant differences among 13 LAB strains, *P* < 0.05.

In order to screen out LAB strains with strong antioxidant capacity, the antioxidant capacity of 12 LAB strains was determined. The antioxidant capacity of LP and PP strains was universally higher than that of LM strains (Figure 1). LP19 and LP26 showed a DPPH radical scavenging ability of 627.12 μg Trolox/ml and 626.02 μg Trolox/ml, respectively; PP18 exhibited the strongest radical scavenging ability at 675.40 μg Trolox/ml (*P* < 0.05). Similar trends were observed in the ABTS and FRAP methods, consistent with the results obtained from the DPPH method. LP26 showed the highest ABTS value at 223.62 μg Trolox/ml, followed by PP25, while SB87 had a lower ABTS value at 115.73 μg Trolox/ml. In terms of FRAP values, LP26 had the highest value at 0.68 μmol Trolox/ml, followed by PP18 with a value of 0.61 μmol Trolox/ml. Based on their antioxidant capacities and absorbance at 600 nm and the pH value of the culture at 24 h (Supplementary Table S1), LP26, PP18, and SB87 were selected as the potential probiotic candidates for enhancing the antioxidant capacity of mulberry silage.

### 3.2 Chemical characteristics of fresh whole plant mulberry

The chemical characteristics of fresh whole mulberry plants as important conditions for starting fermentation are listed in Table 1. DM content was 35.34%, while that of CP and WSC was 14.65% DM and 11.32% DM, respectively. The contents of NDF and ADF were 53.53% DM and 35.99% DM, respectively. A lower level of epiphytic LAB count (4.79 log10 CFU/g FW) was observed, and yeast and coliform counts were 4.01log10 CFU/g FW and 6.23log10 CFU/g FW, respectively.

**Table 1:** Characteristics of the fresh whole plant of mulberry.

**Items**	**Contents**
DM (%)	35.34
CP (% DM)	14.65
WSC (% DM)	11.32
NDF (% DM)	53.53
ADF (% DM)	35.99
LAB (log_10_ CFU/g FW)	4.79
Yeast (log_10_ CFU/g FW)	4.01
Coliform (log_10_ CFU/g FW)	6.23

DM, dry matter; FW, fresh weight; CP, crude protein; WSC, water-soluble carbohydrates; NDF, neutral detergent fiber; ADF, acid detergent fiber; and LAB, lactic acid bacteria.

### 3.3 Chemical characteristics and microbial counts of mulberry silage

Chemical characteristics of mulberry silage that was inoculated with or without *L. plantarum* or *P. pentosaceus* alone, respectively, or in combination with *S. bovis* were determined after 60-day fermentation, as shown in Table 2. Inoculations of LAB significantly increased DM content (*P* < 0.01) and significantly decreased pH (*P* < 0.01) compared with the control group (DM contents 26.70%, pH = 5.30), particularly in the LP-(DM 32.03%, pH = 4.44) and LP_PP_SB-treated groups (DM 31.68%, pH = 4.26). The CP content exhibited the highest in the LP_PP-treated group (CP 14.46% DM, *P* < 0.05). Inoculations of LAB significantly decreased the concentration of AN compared with the control group (5.03 g/kg FM), particularly in the LP_PP_SB-treated group (1.05 g/kg FM, *P* < 0.01), and the concentration of AN of the LP_SB-, PP_SB-, and LP_PP_SB-treated groups in combination with SB significantly decreased compared with that of the LP-, PP-, and LP_PP-treated groups without SB, respectively. The lowest concentration of WSC was observed in the PP-treated groups (4.33% DM, *P* < 0.01), with no significant differences among other treated groups. No significant difference in NDF was observed among all groups. Inoculations of LAB significantly decreased the content of ADF (22.94–27.98% DM *P* < 0.01) compared with the control group (32.89% DM). The concentration of lactic acid was highest in the LP- (44.80 g/kg DM) and LP_PP_SB (46.47 g/kg DM)-treated groups, followed by the PP_SB (18.67 g/kg DM)-treated group. The concentration of acetic acid was the highest in the LP_PP (18.95 g/kg DM)-treated group, followed by the PP- and LP_SB-treated groups. There was no significant difference between the treated groups and the control group. The LP_PP_SB (7.44 log10 cfu/g)-treated group showed the highest populations of LAB and the lowest population of yeast (*P* < 0.01). The populations of coliform in the LP- and LP_PP_SB-treated groups were <2.0. Therefore, *L. plantarum* and *P. pentosaceus*, whether used alone or in combination, were found to improve the fermentation quality of mulberry silage. However, when inoculated alone, *S. bovis* did not improve the quality of mulberry silage.

**Table 2:** Characteristics of whole plant mulberry silage 60 days after inoculation with LAB.

**Items**	**Treatments** ^**a**^								**SEM**	***P***
	**CON**	**SB**	**PP**	**LP**	**LP_PP**	**LP_SB**	**PP_SB**	**LP_PP_SB**		
DM (%)	26.70 ± 0.21^d^	29.90 ± 0.73^bc^	29.81 ± 0.43^c^	32.03 ± 0.31^a^	31.06 ± 1.34^abc^	31.60 ± 1.63^ab^	31.26 ± 0.89^abc^	31.68 ± 0.46^a^	0.002	< 0.01
pH	5.30 ± 0.06^a^	4.66 ± 0.01^b^	4.70 ± 0.08^b^	4.44 ± 0.04^cd^	4.75 ± 0.08^b^	4.77 ± 0.21^b^	4.58 ± 0.10^bc^	4.26 ± 0.01^e^	0.018	< 0.01
CP (% DM)	9.65 ± 0.86^f^	11.74 ± 0.66^e^	11.8 ± 0.70^e^	12.67 ± 0.18^bcd^	14.46 ± 0.14^a^	12.25 ± 0.17^cde^	11.96 ± 0.63^bc^	11.54 ± 0.60^e^	0.079	< 0.01
Ammonia-N (g/kg FM)	5.03 ± 0.10^a^	5.34 ± 0.32^a^	4.71 ± 0.41^a^	3.96 ± 0.15^b^	3.45 ± 0.30^b^	1.86 ± 0.51^cd^	2.27 ± 0.30^c^	1.05 ± 0.45^d^	0.394	< 0.01
WSC (% DM)	5.61 ± 0.22^a^	5.17 ± 0.43^ab^	4.33 ± 0.12^b^	4.53 ± 0.14^ab^	4.91 ± 1.06^ab^	4.81 ± 0.96^ab^	5.62 ± 0.40^a^	5.34 ± 0.30^ab^	0.003	< 0.01
NDF (% DM)	50.05 ± 0.99	42.27 ± 1.97	40.73 ± 4.14	43.57 ± 0.72	43.91 ± 3.05	47.14 ± 7.47	43.25 ± 4.67	32.19 ± 7.38	1.161	0.205
ADF (% DM)	32.89 ± 1.60^a^	26.14 ± 2.79^bc^	26.07 ± 3.15^bc^	27.98 ± 1.86^ab^	24.03 ± 1.62^bc^	26.27 ± 3.37^bc^	25.6 ± 2.30b^c^	22.94 ± 5.86^bc^	0.498	< 0.01
Lactic acid (g/kg DM)	8.88 ± 0.57^b^	15.82 ± 4.66^b^	10.69 ± 5.91^b^	44.80 ± 5.24^a^	9.76 ± 4.82^b^	8.58 ± 6.30^b^	18.67 ± 15.00^b^	46.47 ± 1.77^a^	1.729	< 0.01
Acetic acid (g/kg DM)	7.24 ± 1.75^e^	15.43 ± 2.17^abcd^	17.14 ± 2.10^abc^	16.5 ± 4.07^abc^	18.95 ± 0.91^a^	16.98 ± 1.76^abc^	11.81 ± 1.84^d^	13.21 ± 4.53^cf^	0.383	< 0.01
Propionic acid (g/kg DM)	1.60 ± 0.15	2.79 ± 2.62	2.32 ± 1.25	2.72 ± 1.84	1.15 ± 0.36	1.33 ± 0.34	1.1 ± 0.35	4.1 ± 1.80	0.273	0.144
Butyric acid (g/kg DM)	ND	ND	ND	ND	ND	ND	ND	ND	_	_
LAB (log_10_ CFU/g FW)	5.91 ± 0.08^c^	5.80 ± 0.28^c^	6.99 ± 0.09^b^	7.10 ± 0.10^b^	7.07 ± 0.33^b^	7.18 ± 0.77^ab^	7.05 ± 0.65^b^	7.44 ± 0.19^a^	0.037	< 0.01
Yeast (log_10_ CFU/g FW)	7.87 ± 0.13^a^	6.94 ± 0.13^ab^	6.60 ± 0.19^ab^	5.89 ± 1.24^b^	6.46 ± 0.13^b^	6.49 ± 0.30^ab^	6.01 ± 0.49^b^	5.62 ± 0.42^a^	0.107	< 0.01
Coliform (log_10_ CFU/g FW)	5.03 ± 0.35	4.26 ± 0.49	2.34 ± 0.01	< 2.00	2.45 ± 0.12	2.65 ± 0.03	2.78 ± 0.14	< 2.00	_	_

^*a*^n = 4 CON, untreated control; SB, S. bovis; LP, L. plantarum; PP, P. pentosaceus; LP_PP, L. plantarum + P. pentosaceus, inoculation ratio of 1:1; LP_SB, L. plantarum + S. bovis, inoculation ratio of 1:1; PP_SB, P. pentosaceus + S. bovis, inoculation ratio of 1:1; LP_PP_SB, L. plantarum + P. pentosaceus + S. bovis, inoculation ratio of 1:2:1, same as others. Different lowercase letters indicate significant differences (*P* < 0.01) among treatment groups.

### 3.4 The antioxidant capacity of silage

**Figure 2:**
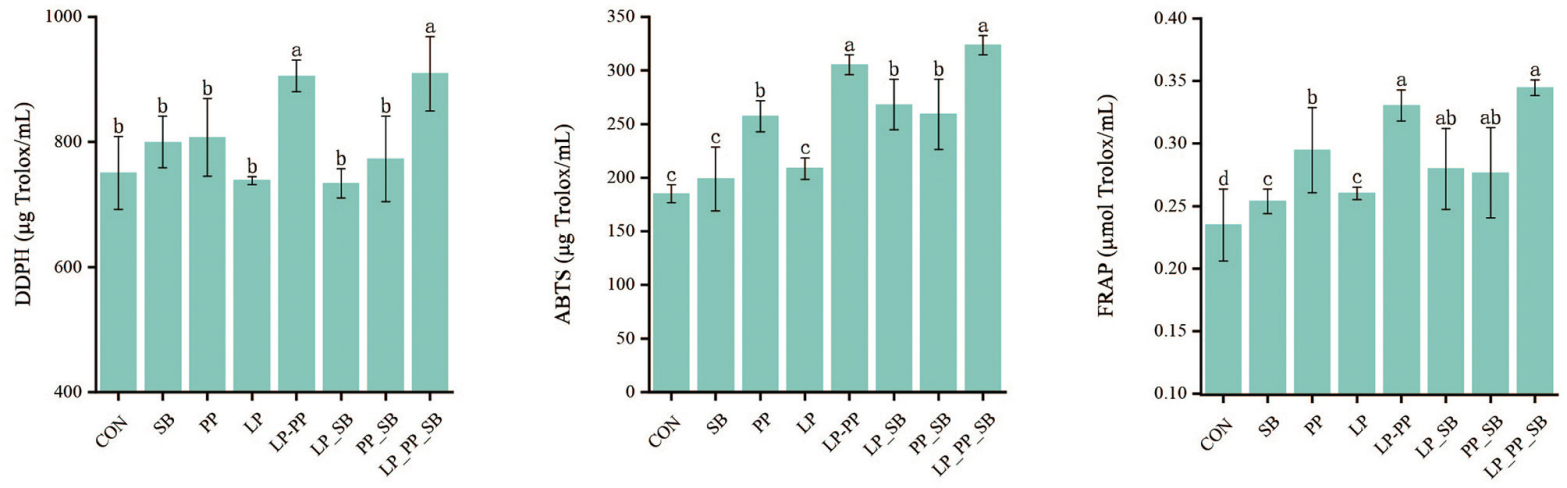
Antioxidant capacity of mulberry silage. Different lowercase letters on the bar represent statistically significant differences among groups, *P* < 0.05.

The antioxidant capacity of mulberry silage inoculated with *L. plantarum* or *P. pentosaceus* with higher antioxidant capacity alone, respectively, or in combination with *S. bovis* was determined by DDPH, FRAP, and ABTS methods after fermentation for 60 days (Figure 2). DPPH in the LP_PP- and LP_PP_SB-treated groups was found to be the highest with 905.42 μg Trolox/ml and 909.04 μg Trolox/ml, respectively, than the control group (750.35 μg Trolox/ml) and other treated groups (733.56–807.31 μg Trolox/ml *P* < 0.05); similarly, ABTS was the highest in the LP_PP- and LP_PP_SB-treated groups of 305.35 μg Trolox/ml and 323.75c, respectively, followed by the PP- (257.51 μg Trolox/ml), LP_SB- (268.16 μg Trolox/ml), and PP_SB (259.06 μg Trolox/ml)-treated groups. Additionally, FRAP was the highest in the LP_PP- and LP_PP_SB-treated groups of 0.33 μmol Trolox/ml and 0.34 μmol Trolox/ml, respectively, followed by the LP_SB- (0.28 μmol Trolox/ml) and PP_SB (0.28 μmol Trolox/ml)-treated groups. Therefore, inoculants of LAB with higher antioxidant capacity improved mulberry silage compared with the control group, particularly in the LP_PP- and LP_PP_SB-treated groups.

### 3.5 Fatty acid composition of mulberry ensiled for 60 days

To our knowledge, fatty acid composition of mulberry silage was determined for the first time (Table 3). Inoculated LAB (except for the SB-treated group) increased the fatty acid content. The saturated fatty acid (SFA) content of six treated groups (1.9583–2.0966 g/kg DM) was higher than that of the control group (1.197 g/kg DM) and the SB (1.1276 g/kg DM)-treated group. There was no significant difference in monounsaturated fatty acid (MUFA) contents. PUFA content was the highest in the LP_PP_SB (1.2851 g/kg DM)-treated group than in other inoculation-treated groups (0.9102–1.1137 g/kg DM) and the control group (1.0074 g/kg DM).

**Table 3:** Fatty acid composition (g/kg DM) of mulberry silage fermented for 60 days.

**Fatty acid**	**CON**	**SB**	**PP**	**LP**	**LP_SB**	**PP_SB**	**LP_PP**	**LP_PP_SB**	**SEM**	***P***
C14:0	0.0053 ± 0.0015^b^	0.0048 ± 0.0005^b^	0.0129 ± 0.0032^a^	0.0098 ± 0.0016^a^	0.0112 ± 0.0017^a^	0.0129 ± 0.0030^a^	0.0120 ± 0.0018^a^	0.0128 ± 0.0043^a^	0.007	< 0.05
C14:1	0.0013 ± 0.0007	0.0014 ± 0.0006	0.0013 ± 0.0002	0.0015 ± 0.0020	0.0016 ± 0.0002	0.0013 ± 0.0001	0.0016 ± 0.0003	0.0017 ± 0.0003	0.0026	0.441
C15:0	0.0015 ± 0.0004^b^	0.0017 ± 0.0009^b^	0.0040 ± 0.0005^ab^	0.0043 ± 0.0003^a^	0.0046 ± 0.0004^a^	0.0042 ± 0.0009^a^	0.0038 ± 0.0002^ab^	0.0045 ± 0.0011^a^	0.0017	< 0.01
C16:0	0.7735 ± 0.1998^b^	0.7647 ± 0.2766^b^	1.3361 ± 0.1689^a^	1.2594 ± 0.1779^a^	1.3623 ± 0.1775^a^	1.3361 ± 0.1311^a^	1.3143 ± 0.2337^a^	1.4289 ± 0.4197^a^	0.0407	< 0.05
C16:1	0.0262 ± 0.0039^b^	0.0251 ± 0.0078^b^	0.0389 ± 0.0042^b^	0.0394 ± 0.0081^b^	0.0370 ± 0.0074^b^	0.0377 ± 0.0058^b^	0.0412 ± 0.0034^a^	0.0484 ± 0.0123^a^	0.0203	< 0.05
C17:0	0.0146 ± 0.0020^b^	0.0129 ± 0.0011^b^	0.0298 ± 0.0048^a^	0.0270 ± 0.0037^a^	0.0293 ± 0.0012^a^	0.0302 ± 0.0064^a^	0.0269 ± 0.0065^a^	0.0322 ± 0.0087^a^	0.0127	< 0.01
C18:0	0.1835 ± 0.0454^b^	0.1890 ± 0.0397^b^	0.3420 ± 0.0521^a^	0.3343 ± 0.0263^b^	0.3746 ± 0.0483^a^	0.3483 ± 0.0449^b^	0.3484 ± 0.0589^a^	0.3785 ± 0.0856^a^	0.1421	< 0.01
C18:1n9c	0.0536 ± 0.0054^b^	0.0537 ± 0.0039^b^	0.0941 ± 0.0155^a^	0.0762 ± 0.0142^ab^	0.0899 ± 0.0148^ab^	0.0733 ± 0.0122^ab^	0.0672 ± 0.011^ab^	0.0962 ± 0.0022^a^	0.0403	< 0.05
C18:2n6c	0.3772 ± 0.0171^b^	0.3671 ± 0.031^b^	0.4547 ± 0.0196^ab^	0.3976 ± 0.0334^b^	0.4035 ± 0.0308^b^	0.3566 ± 0.0181^b^	0.3706 ± 0.556^b^	0.5103 ± 0.0802^a^	0.1107	< 0.01
C20:0	0.049 ± 0.0069^b^	0.0496 ± 0.0020^b^	0.0804 ± 0.0065^a^	0.0800 ± 0.0039^a^	0.0890 ± 0.0067^a^	0.0816 ± 0.0091^a^	0.0741 ± 0.0033^a^	0.0900 ± 0.0197^a^	0.0269	< 0.01
C18:3n3	0.5616 ± 0.1172	0.5076 ± 0.0626	0.6187 ± 0.1025	0.4920 ± 0.0763	0.5981 ± 0.0874	0.5667 ± 0.0920	0.5743 ± 0.0999	0.7272 ± 0.1990	0.3211	0.259
C21:0	0.099 ± 0.0005^c^	0.0106 ± 0.0009^bc^	0.0140 ± 0.0035^abc^	0.0125 ± 0.0007^abc^	0.0140 ± 0.0008^ab^	0.0144 ± 0.0007^ab^	0.0142 ± 0.0021^ab^	0.0147 ± 0.0026^a^	0.0053	< 0.05
C20:2	0.0032 ± 0.0001	0.0032 ± 0.0003	0.0036 ± 0.0008	0.0041 ± 0.0001	0.0046 ± 0.0008	0.0039 ± 0.0006	0.0042 ± 0.0003	0.0047 ± 0.0007	0.0017	0.113
C22:0	0.0505 ± 0.0083^b^	0.0489 ± 0.0050^b^	0.0728 ± 0.0043^a^	0.0756 ± 0.0049^a^	0.0765 ± 0.0037^a^	0.0768 ± 0.0028^a^	0.0738 ± 0.0015^a^	0.0770 ± 0.0107^a^	0.0179	< 0.01
C20:3n3	0.0226 ± 0.0053^b^	0.0208 ± 0.0025^b^	0.0244 ± 0.0029^b^	0.0225 ± 0.0038^b^	0.0303 ± 0.0036^a^	0.0205 ± 0.0020^b^	0.0283 ± 0.0067^ab^	0.0338 ± 0.0060^a^	0.00117	< 0.05
C22:1n9	0.2116 ± 0.0592	0.1908 ± 0.0073	0.2164 ± 0.0157	0.1838 ± 0.0389	0.0191 ± 0.0635	0.1856 ± 0.0339	0.2558 ± 0.0475	0.2002 ± 0.0285	0.1163	0.783
C23:0	0.0120 ± 0.0009^b^	0.0119 ± 0.0008 ^ab^	0.015 ± 0.0005^ab^	0.0190 ± 0.0043^a^	0.0169 ± 0.0027^a^	0.0139 ± 0.0022^ab^	0.0144 ± 0.0008^ab^	0.0168 ± 0.0049^ab^	0.0084	< 0.05
C20:5n3	0.0144 ± 0.0020	0.0146 ± 0.0024	0.0159 ± 0.0023	0.0133 ± 0.0013	0.0144 ± 0.0015	0.0157 ± 0.0010	0.0128 ± 0.0022	0.0138 ± 0.0026	0.0053	0.573
C24:0	0.030 ± 0.0019	0.0299 ± 0.0017	0.0359 ± 0.0027	0.0327 ± 0.0022	0.0356 ± 0.0045	0.0316 ± 0.0064	0.0311 ± 0.0033	0.0367 ± 0.0081	0.0118	0.411
SFA	1.197 ± 0.2647^b^	1.1276 ± 0.3130^b^	1.9583 ± 0.2517^a^	1.8585 ± 0.2123^a^	2.0315 ± 0.2459^a^	1.9549 ± 0.1808 ^a^	1.9193 ± 0.2910^a^	2.0966 ± 0.5650^a^	0.8628	< 0.05
MUFA	0.3113 ± 0.0687	0.2709 ± 0.0111	0.3506 ± 0.0273	0.3009 ± 0.0216	0.3666 ± 0.0492	0.2981 ± 0.0251	0.3644 ± 0.0340	0.3465 ± 0.0629	0.1286	0.346
PUFA	1.0074 ± 0.1330^b^	0.9102 ± 0.0731^b^	1.1137 ± 0.1220^b^	0.9253 ± 0.1034^b^	0.9531 ± 0.1167^b^	0.9596 ± 0.1038^b^	0.9856 ± 0.1386^b^	1.2851 ± 0.2877^a^	0.4317	< 0.05

Different lowercase letters indicate significant differences (P < 0.05) among treatment groups.

### 3.6 Bacterial community of mulberry ensiled for 60 days

The bacterial community plays a crucial role in unraveling the intricate relationship between inoculants of LAB and the overall quality of mulberry silage fermentation. The alpha diversity of the bacterial community in mulberry silage for 60 days is shown in Table 4. Coverage values >0.99 were obtained for all samples. The Shannon index was higher in inoculation-treated groups (except for the SB-treated group), while the Simpson index showed the inverse of the Shannon index. This finding indicated that inoculants of LABs decrease the biological diversity of mulberry silage.

**Table 4:** Alpha diversity of the bacterial community of mulberry silage.

**Items**	**CON**	**SB**	**LP**	**PP**	**LP_PP**	**LP_SB**	**PP_SB**	**LP_PP_SB**
Ace	120.5 ± 8.45	143.3 ± 24.37	155.0 ± 28.26	110.8 ± 18.64	111.8 ± 19.80	131.2 ± 28.02	119.5 ± 22.67	115.1 ± 11.75
Chao	118.2 ± 6.49	132.5 ± 23.53	138.0 ± 18.35	107.7 ± 18.07	105.4 ± 16.68	108.5 ± 16.75	103.6 ± 13.19	101.9 ± 13.17
Sobs	103.0 ± 7.85	102.0 ± 21.08	110.8 ±12.57	84.3 ± 24.24	89.5 ± 13.99	85.0 ± 13.84	71.3 ± 10.96	81.3 ± 9.91
Shannon	1.905 ± 0.098^a^	1.960 ± 0.204^a^	0.839 ± 0.473^b^	1.105 ± 0.318^b^	0.683 ± 0.407^b^	0.863 ± 0.249^b^	0.670 ± 0.236^b^	0.761 ± 0.045^b^
Simpson	0.264 ± 0.261^b^	0.245 ± 0.052^b^	0.696 ± 0.180^a^	0.584 ± 0.121^a^	0.725 ± 0.209^a^	0.682 ± 0.105^a^	0.744 ± 0.134^a^	0.602 ± 0.152^a^
Coverage	0.9995 ± 0.00008	0.9994 ± 0.00010	0.9992 ± 0.00013	0.9995 ± 0.0004	0.9996 ± 0.0007	0.9994 ± 0.0013	0.9995 ± 0.0008	0.9994 ± 0.0006

Different lowercase letters indicate significant differences (P < 0.05) among treatment groups.

**Figure 3:**
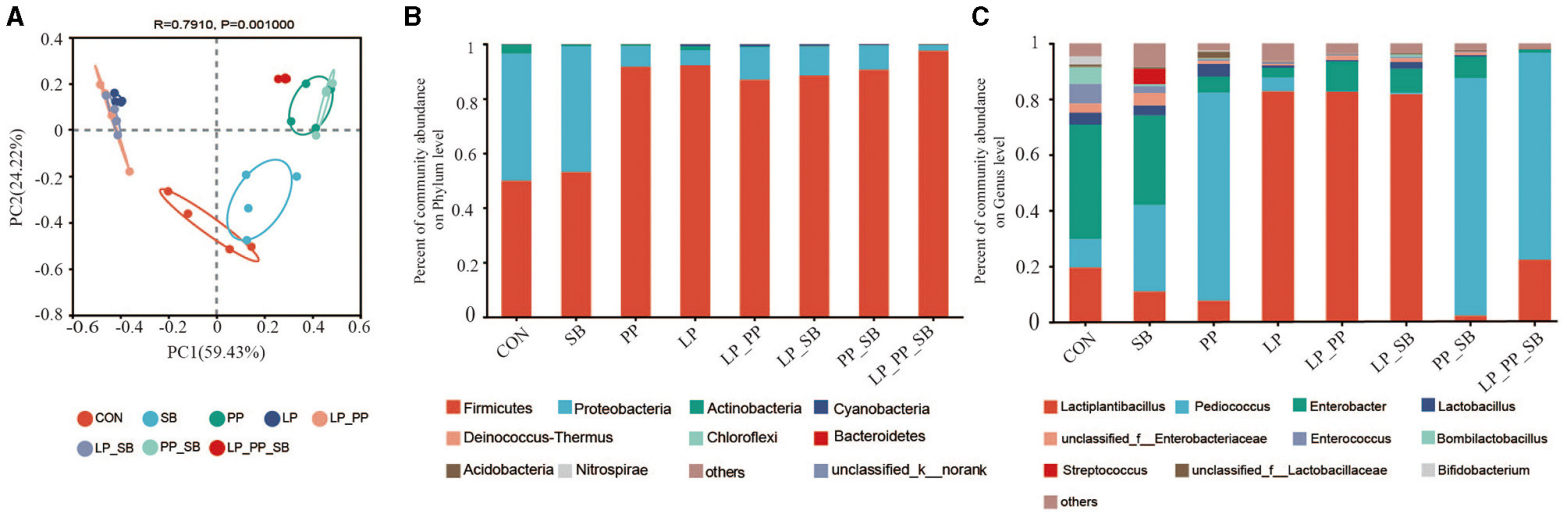
Principal coordinate analysis (PCoA) on the operational taxonomic unit (OTU) level of mulberry silage **(A)**; bacterial community analysis of mulberry silage at the phylum **(B)** and the genus level **(C)**.

The results of the principal component analysis (PCoA) of the operational taxonomic units (OTUs) of mulberry silage are shown in Figure 3A (R = 0.79, *P* = 0.001). The LP_PP_SB-, PP-, and PP_SB-treated groups were closely clustered together, and the LP_PP-, LP-, and LP_SB-treated groups were also tightly clustered together. All these groups were clearly distant from the control and the SB-treated groups.

At the phylum level (Figure 3B), *Firmicutes* and *Proteobacteria* were dominant in the control group (50.96% and 45.85%, respectively) and the SB-treated group (58.24% and 40.89%, respectively). *Firmicutes* (88.21–97.59%) dominated in the other six treated groups, followed by *Actinobacteria* (0.09–1.52%) and *Cyanobacteria* (0.06–0.73%). At the genus level (Figure 3C), *Enterobacter* was dominant in the control and SB-treated groups, and the relative abundance was 41.04% and 32.15%, respectively, followed by *Pediococcus* and *Lactiplantibacillus. Lactiplantibacillus* was dominant in the LP-, LP_PP-, and LP_SB-treated group, with a relative abundance of 82.27%, 82.69%, and 81.84%, respectively, *P* < 0.05), followed by *Enterobacter*. Relative abundance of *Pediococcus* was dominant in the PP-, PP_SB-, and LP_PP_SB-treated groups (74.67%, 85.27%, and 74.27%, respectively, *P* < 0.05). Relative abundance of *Streptococcus* (0.04–5.56%) was not dominant in the SB-, LP_SB-, PP_SB-, and LP_PP_SB-treated groups inoculated with *S. bovis*.

### 3.7 Correlation analysis of the characteristics and bacterial community of mulberry silage

**Figure 4:**
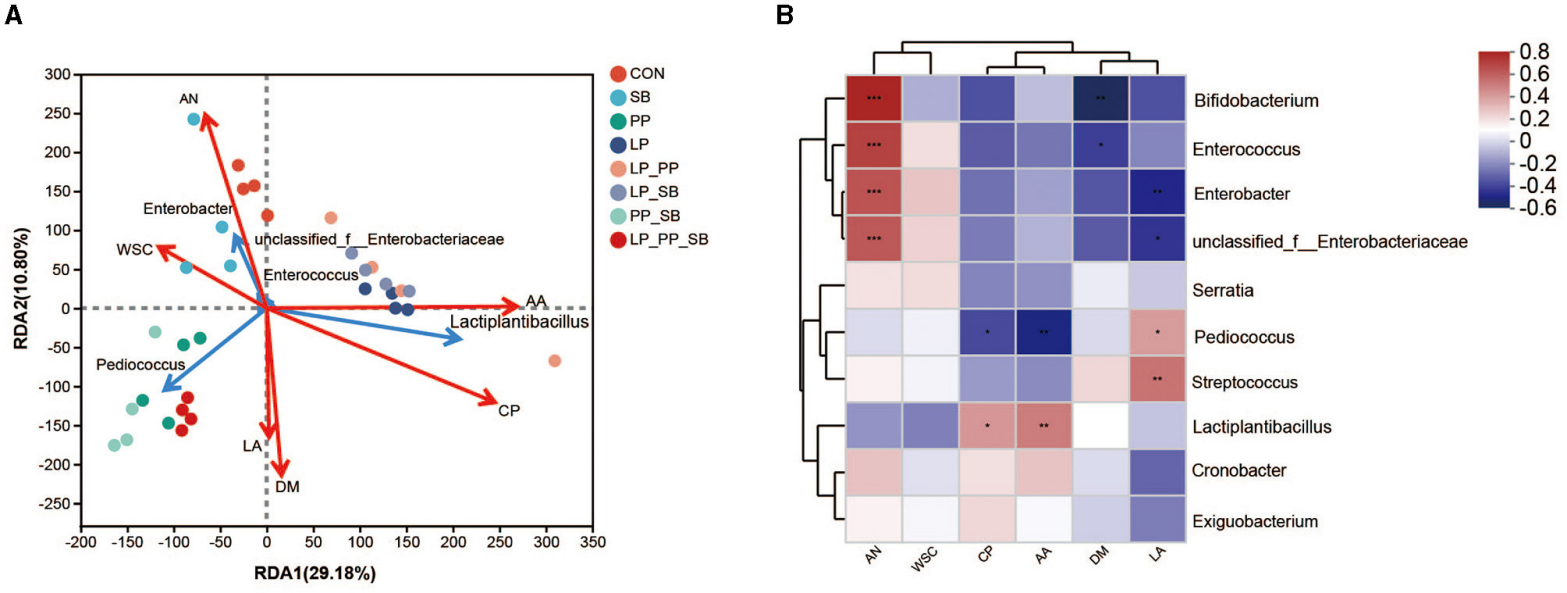
Redundancy analysis of bacteria and chemical characteristics in mulberry silage **(A)**; the heat map of the correlation between bacteria and chemical characteristics in mulberry silage **(B)**. *P < 0.05, **P < 0.01, and ***P < 0.001.

Redundancy analysis (RDA) and a heat map revealed a relationship between the relative abundance of the top 5 bacteria and the top 10 bacteria and the chemical characteristics of mulberry silage at 60 days (Figures 4A, B). The results of RDA showed that DM, LA, CP, and AA contents were positively correlated with *Pediococcus* and *Lactiplantibacillus*, while AN and WSC contents were positively correlated with *Enterobacter* (Figure 4A). The results of the heat map showed that AN content was positively correlated with *Enterobacter* (R = 0.54, *P* < 0.01), while CP and AA contents were positively correlated with *Lactiplantibacillus* (R = 0.42, *P* < 0.05, R = 0.61, *P* < 0.01, respectively). DM was negatively corrected with *Enterobacter* (R = 0.54, *P* < 0.01).

The concentration of LA was significantly positively correlated with *Lactiplantibacillus* (R = 0.37, *P* < 0.05), but significantly negatively correlated with *Enterobacter* (R = −0.45, *P* < 0.01). The concentrations of AA and CP were positively correlated with *Lactiplantibacillus* (R = 0.46, *P* < 0.01, and R = 0.38, *P* < 0.05, respectively), but negatively correlated with *Pediococcus* (R = −0.47, *P* < 0.01, and R = −0.36, *P* < 0.05, respectively). The DM content was negatively correlated with *Enterococcus* abundance (R = −0.39, *P* < 0.05), while AN content was significantly and positively correlated with *Enterobacter* (R = 0.58, *P* < 0.001) and *Enterococcus* (R = 0.63, *P* < 0.001).

### 3.8 Effect of inoculation of LAB on mulberry silage metabolites

#### 3.8.1 Metabolite profile of mulberry silage

**Figure 5:**
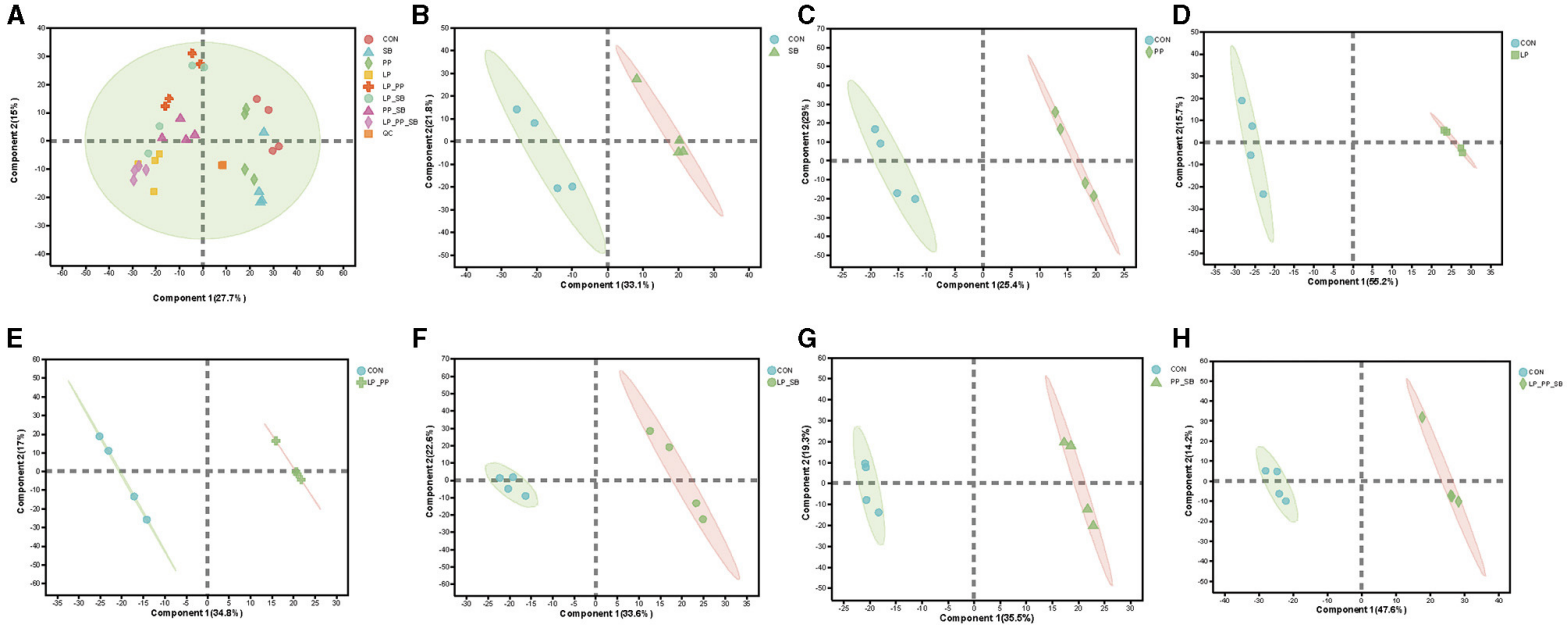
Metabolomics analysis of mulberry silage after fermentation for 60 days. PLS-DA score plot results from metabolites of the CON-, SB-, PP-, LP-, LP_PP-, LP_SB-, PP_SB-, and LP_PP_SB-treated groups **(A)**, PLS-DA score plots of metabolites between the control and each treated group **(B–H)**.

Inoculants of LAB with higher antioxidant capacity would alter metabolites of mulberry silage by different metabolite pathways. A total of 1409 metabolites were identified through untargeted metabolomics analysis in whole groups after fermentation for 60 days. Among them, there were 835 metabolites of the negative mode and 574 metabolites of the positive mode. There was a clear clustering phenomenon among samples of each LP-, LP_PP-, LP_SB-, PP_SB-, and PP_SB-treated groups that exhibited a significant distance from the control group, while the SB- and PP-treated groups were closer in distance to the control group (Figure 5A). PLS-DA analysis between the control and each treated groups showed that inoculation of LABs changed metabolites from the control group (Figures 5B–H). Permutation testing was conducted on the PLS_DA model, indicating no overfitting phenomenon in the model (Supplementary Figure S2).

**Figure 6:**
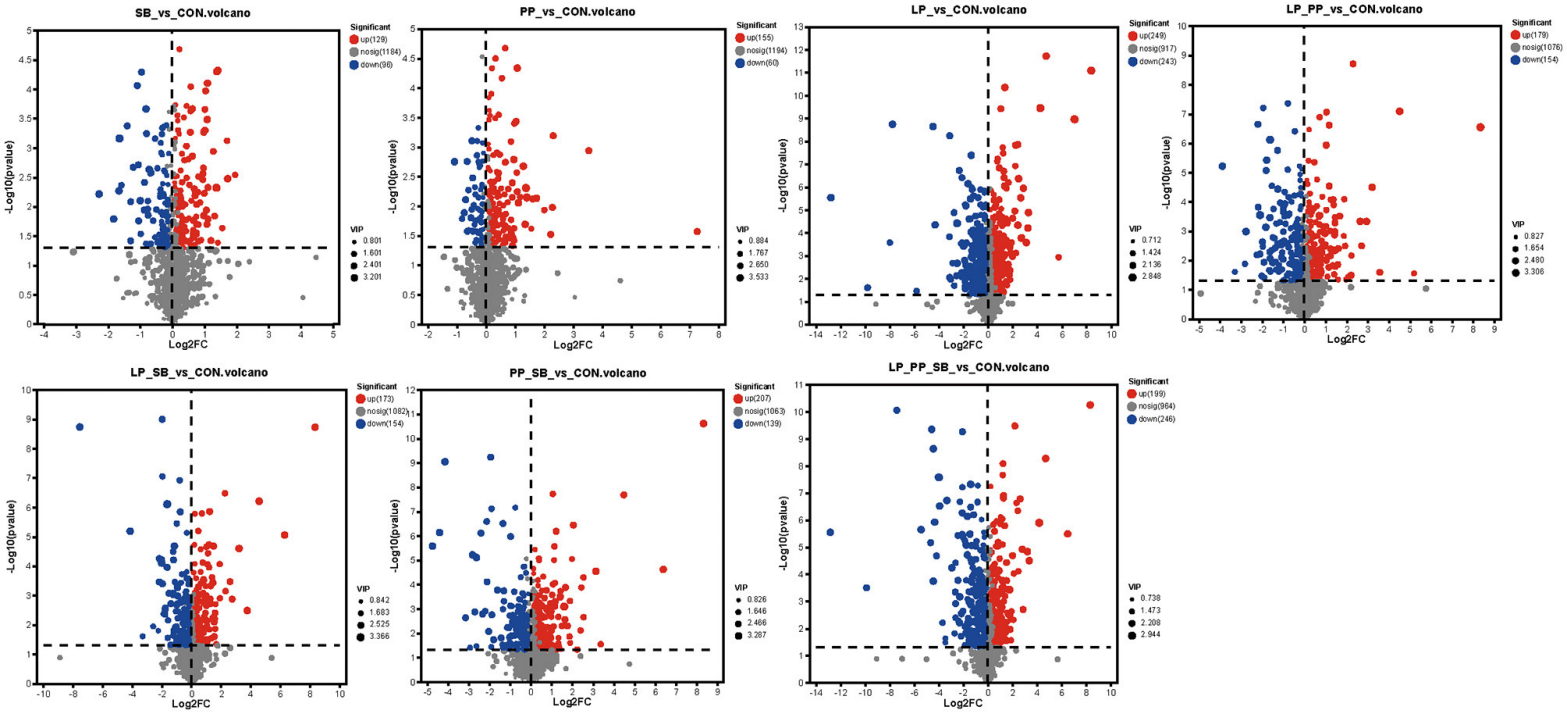
Volcano of different metabolites between each treated group and control.

Differences between metabolites between the control group and each group were statistically analyzed by Student's *t*-test and two-tailed test (Figure 6). Among them, the LP-treated group showed the highest number of differential metabolites, with 249 upregulated and 243 downregulated, followed by the LP_PP_SB- and PP_SB-treated group, with 199 and 207 upregulated and 246 and 139 down regulated, respectively. These were consistent with the results of PLS-DA analysis (Figure 5A) that LP- and LP_PP_SB-treated groups showed more different metabolites compared to the control group.

**Figure 7:**
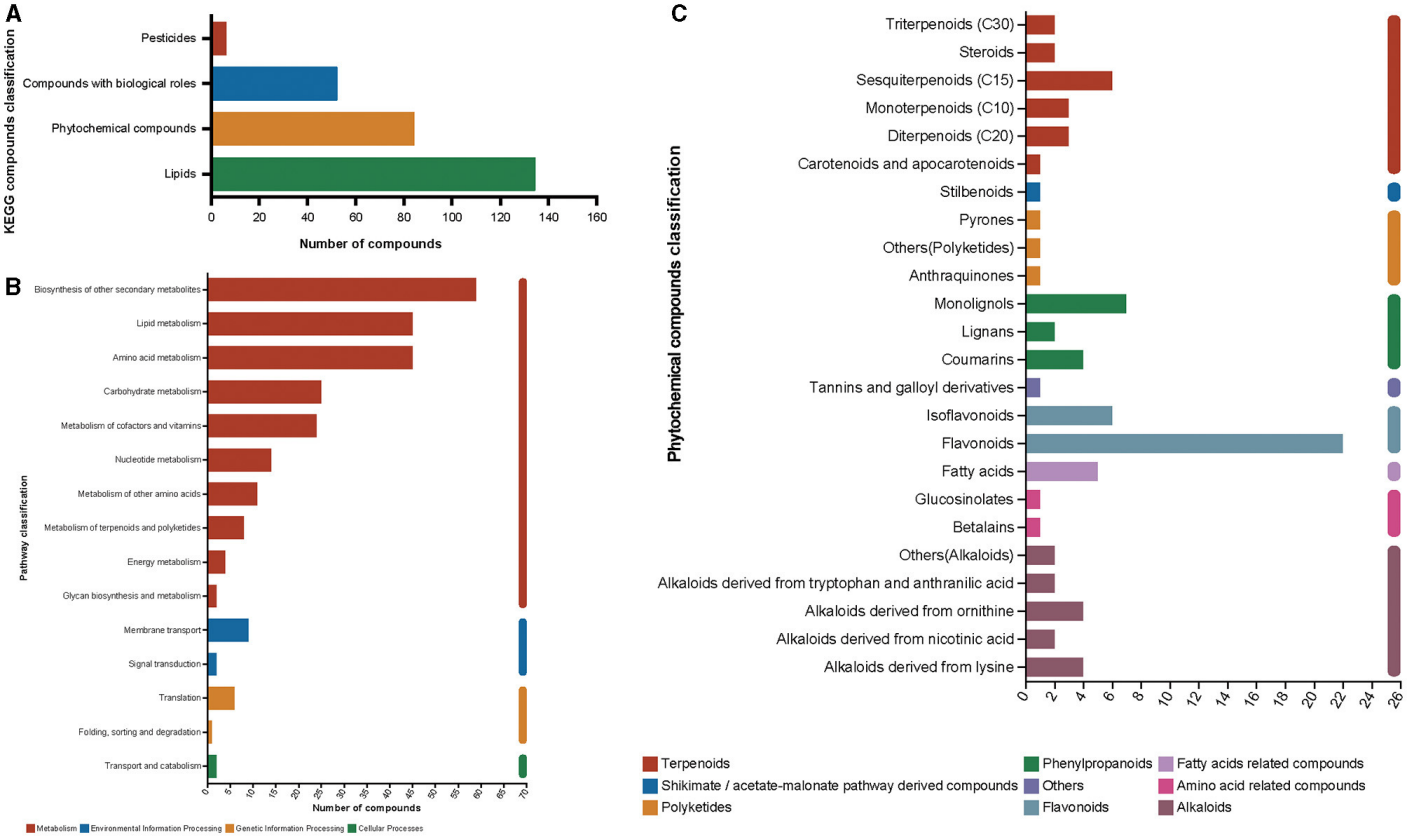
KEGG compound classification of metabolites after fermentation for 60 days **(A)**, pathway classification of metabolites after fermentation for 60 days **(B)**, and phytochemical compound classification of KEGG compound **(C)**.

Metabolites derived from mulberry silage after fermentation for 60 days could be categorized into four groups of KEGG compounds: pesticides, bioactive compounds, phytochemicals, and lipids. Among these classifications, lipids exhibit the highest abundance with a total of 134 compounds, followed by phytochemicals that encompass 84 compounds (Figure 7A). In functional pathway classification, biosynthesis of other secondary metabolites was found to have the highest number of annotated compounds (59 compounds, Figure 7B). Relevant information on 59 compounds has been presented in Supplementary Table S2. Among phytochemical compounds of annotated categories, flavonoids (including flavonoids and isoflavonoids) accounted for 33.33% of the total metabolites in phytochemical compounds (Figure 7C). These results indicated that compounds related to antioxidant activity account for a high proportion of the metabolites.

#### 3.8.2 Metabolites associated with antioxidant capacity of silage

All the upregulated and downregulated differential metabolites of phytochemical compounds were annotated into the biosynthesis of the secondary metabolite pathway (Supplementary Figure S3), which observed that upregulated and downregulated compounds were distinctly clustered, respectively. Upregulated compounds mainly accumulate in pathways of flavone and flavonol biosynthesis, flavonoid biosynthesis, and lysine biosynthesis.

**Figure 8:**
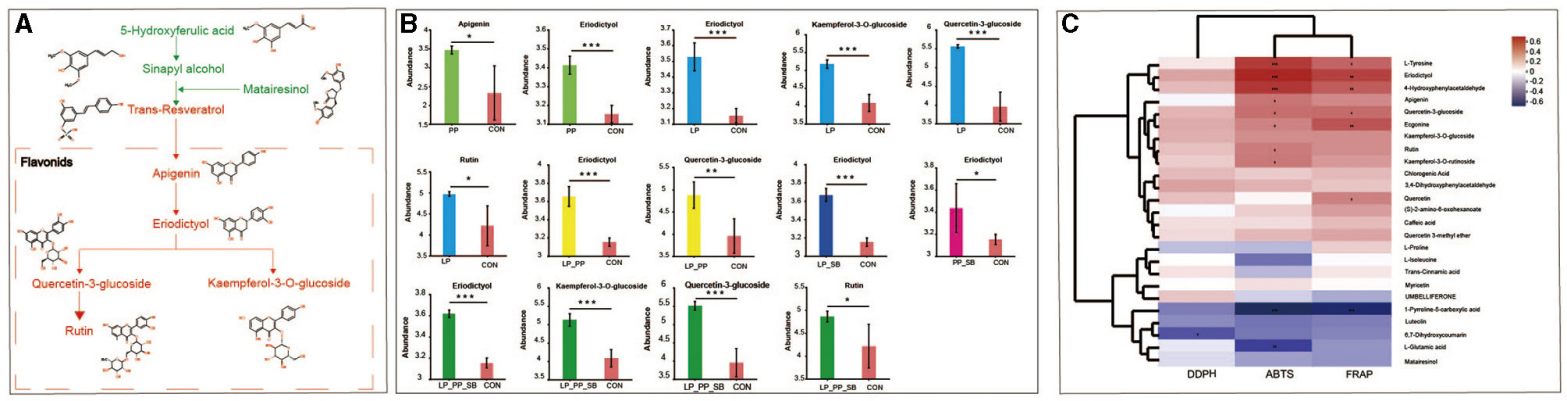
Pathways of nine key metabolite compounds associated with the antioxidant capacity of mulberry silage **(A)**; relative abundance of upregulated five flavonoids in the PP-, LP-, LP_PP-, LP_SB-, PP_SB-, and LP_PP_SB-treated groups and control, respectively **(B)**; Pearson's correlation analysis between the top 25 metabolites of the biosynthesis of secondary metabolite pathway and DPPH, ABTS, and FRAP of mulberry silage **(C)**. ^*^P < 0.05, ^**^P < 0.01, ^***^P < 0.001.

Nine key metabolites associated with antioxidant capacity and compound metabolic pathways are shown in Figure 8A. Among them, 5-hydroxyferulic acid, sinapyl alcohol, and matairesinol were downregulated, while apigenin, eriodictyol, quercetin-3-glucoside, rutin, and kaempferol-3-o-glucoside were upregulated. Among five upregulated groups, eriodictyol was upregulated in LAB-inoculated groups (except for SB) and had higher relative abundance in the LP_PP- and LP_PP_SB-treated groups, with values of 3.65 and 3.61, respectively (Figure 8B and Supplementary Table S3). Apigenin was upregulated in the PP (3.41)-treated group, while kaempferol-3-o-glucoside and rutin were upregulated in the LP- (5.18 and 5.13, respectively) and LP_PP_SB (5.13 and 4.86, respectively)-treated groups. Additionally, quercetin-3-glucoside was upregulated in the LP- (5.57), LP_PP- (4.88), and LP_PP_SB (5.51)-treated groups. The heat map showed that ABTS of silage was significantly positively correlated with four flavonoids, namely, apigenin (R = 9.49, *P* < 0.05), eriodictyol (R = 0.64, *P* < 0.001), quercetin-3-glucoside (R = 0.41, *P* < 0.05), and rutin (R = 0.38, *P* < 0.05), upregulated in the biosynthesis of secondary metabolites pathway, while FRAP was significantly positively correlated with eriodictyol (R = 0.55, *P* < 0.01) and quercetin-3-glucoside (R = 0.55 *P* < 0.01) (Figure 8C). This result is consistent with that of the metabolic pathways of nine compounds (Figure 8A). Among them, only eriodictyol was significantly positively correlated with ABTS or FRAP values of silage.

## 4 Discussion

### 4.1 Antioxidant capacity of LAB strains

Antioxidant capacity showed significant differences between LM, LP, and PP strains, and DDPH, ABTS, and FRAP levels of LM20 were higher than those in other LM strains, indicating that antioxidant capacity exhibits strain-specificity among LAB strains. Similarly, it was previously reported that antioxidant capacity was different among four *L. plantarum* strains (Rwubuzizi et al., 2023). The antioxidant capacity of LAB mainly contributed to exopolysaccharides such as fructose and rhamnose (Pan and Mei, 2010). The differential antioxidant capacity among LAB strains may suggest that there were variations in exopolysaccharide content and composition among different strains.

### 4.2 Characteristics of mulberry silage

Previous studies had indicated that higher dry matter (>35% DM), a sufficient concentration of WSC (>6.0% DM), and sufficient epiphytic LAB (>1.0 × 105log10 CFU/g FW) were necessary factors for ensiling (Herrmann et al., 2015; Oliveira et al., 2017). In the present study, DM (35.34%) and WSC (11.32%) were both suited for ensiling, but epiphytic LAB (4.79log10 CFU/g FW) was < 1.0 × 10^5^log10 CFU/g FW. This indicated that lack of adequate epiphytic LAB was the dominant reason leading to poor quality of mulberry silage (pH = 5.30) (Table 2). Therefore, it was necessary to inoculate LAB for improvement in mulberry silage quality. Results showed that all inoculated treated groups (except for the SB-treated group) had improved quality of mulberry silage, especially in the LP_PP_SB-treated group. Inoculations of LAB reduced DM loss compared to the control group. This was consistent with the observation in a previous study, where *L. plantarum* inoculation reduced DM loss and could promote homo-fermentation, resulting in the inhibition of organic matter degradation during ensiling (Ren et al., 2020). Usually, an LA-to-AA ratio of >3 indicates homo-fermentation, while an LA-to-AA ratio of <3 indicates hetero-fermentation (Ali et al., 2020). In this study, only LP- and LP_PP_SB-treated groups exhibited homo-fermentation during ensiling. Thus, the mechanism of reduction in DM loss in other inoculation groups requires further study. LA contents were the highest in the LP- (44.80 g/kg DM) and LP_PP_SB (46.47 g/kg DM)-treated groups, while other groups showed no difference in LA content (8.58–18.67 g/kg DM). Notably, in the present study, the WSC content was not different among treated groups (except for the PP-treated group), indicating that these groups had the same capacity for WSC utilization. Thus, these results suggest that the ability of LAB to convert WSC to LA was enhanced only when *S. bovis* was combined with *L. plantarum* and *P. pentosaceus*. The WSC content in the PP-treated group was lower than that in the control and other treated groups. A study also observed lower WSC content in Napier grass silage inoculated with *P. pentosaceus* than those in the control group and silage inoculated with *L. plantarum* (Li et al., 2023). These results indicate that inoculation with *P. pentosaceus* has a strong capacity for substrate fermentation.

In the present study, *P. pentosaceus* inoculation resulted in a higher AN content than that of other inoculation groups (*P* < 0.05). Some *Pediococcus* strains can produce proteases in fermented food, in which the enzyme is stable at 20–40°C and shows pH stability between 4.0 and 7.0 (Xu et al., 2015). Theoretically, a pH between 4.26 and 5.30 shows that these proteases remained functional when produced by *Pediococcus*. Further study is needed to determine whether these bacteria secrete proteases during ensiling. Additionally, the inhibition of *Pediococcus* activity partly resulted in lower protein degradation during ensiling, which is attributed to *Pediococcus* having the capacity to facilitate *Enterobacter* activity for amino acid degradation (Huang et al., 2022). Inoculation with *S. bovis* or *L. plantarum* alone decreased AN content compared to the control group (*P* < 0.05). The combination of *S. bovis* with other LAB strains showed a greater capacity to inhibit AN production, particularly when *L. plantarum, P. pentosaceus*, and *S. bovis* were combined. Generally, *Enterobacter* and *Clostridium* are involved in AN production during ensiling (Pahlow et al., 2015). The present study did not detect BA, indicating the lack of *Clostridium* fermentation during ensiling. Thus, *Enterobacter* may be the main bacterium responsible for AN production. *Enterobacter* was inhibited when the pH was lower than 4.5 (Huang et al., 2022). In the present study, the pH of the control and the PP-, SB-, LP_SB-, and PP_SB-treated groups was over 4.5 after 60 days of ensiling, and no significant differences were observed among the inoculation groups (*P* > 0.05). From these results, it can be assumed that there was no difference in AN content between the PP, SB, LP_SB, and PP_SB-treated groups. However, these groups showed lower AN content, particularly in the *P. pentosaceus* combined with the *S. bovis*-inoculated groups. Inoculation in the LP and LP_PP_SB-treated groups resulted in a pH below 4.5, thereby inhibiting AN production. These results suggest that inoculation with SB and other LAB strains can inhibit AN production. Notably, this effect was not only impacted by pH; however, the mechanism underlying this impact requires further study.

### 4.3 Antioxidant capacity of silage

The present study demonstrated that FRAP levels significantly increased in all treated groups inoculated with LAB strains compared with the control group (Figure 2). Furthermore, the highest levels of DDPH, ABTS, and FRAP levels were observed in the LP_PP- and LP_PP_SB-treated groups. This finding suggested that inoculations of LAB were beneficial to improvement in the antioxidant capacity of mulberry silage, particularly when *L. plantarum* was combined with *P. pentosaceus*. According to Huynh et al. (2014), LABs were capable of converting bound phenolic compounds in plants, such as cellulose, hemicellulose, lignin, pectin, and proteins. They can also transform these phenolic compounds into different metabolites, thereby exhibiting various biological activities in fermented foods. It has been established that both the *L. plantarum* and *P. pentosaceus* enhance the antioxidant capacity of fermentations, such as *Momordica charantia* juice, alfalfa, or Yak-Kong soybean, by increasing the content and altering compositions of bioactive compounds (Gao et al., 2019; Li et al., 2021), which aligns with the findings of our research. These transformations were facilitated by the activity of various enzymes from LABs, including glycosyl hydrolases, reductases, and esterases (Pasquale et al., 2018). Interestingly, the antioxidant capacity of mulberry silage varied among seven treated groups, which may be due to the strain specificity of LAB in terms of enzyme activity, resulting in different efficiencies in the release or transformation of phenolic compounds across different treated groups. The higher antioxidant capacity in the LP_PP- and LP_PP_SB-treated groups suggested that certain LAB enzyme activities related to the release or transformation of phenolic compounds were intensified, leading to the accumulation of more compounds with antioxidant activity when *L. plantarum* was combined with *P. pentosaceus*. Although the enzymes were not qualitatively and quantitively analyzed in our study, these results preliminarily suggested that the combination of *L. plantarum* and *P. pentosaceus* could serve as an effective LAB inoculant for improving the antioxidant capacity of mulberry silage.

### 4.4 Fatty acid composition of silage

To the best of our knowledge, the fatty acid composition of mulberry silage was first qualitatively and quantitively analyzed in the present study. Contents of SFA, MUFA, and PUFA were less than those of alfalfa silage (Zhang et al., 2021), which contributed to a variety of silage raw materials. In the present study, the LP_PP_SB-treated group showed the highest content of PUFAs due to the increase in C18:1n9c and C18:2n6c. Fatty acids could be biohydrogenated by lipoxygenases from plants during fermentation (Zhang et al., 2021). The activity of lipoxygenases was highly sensitive to pH, with an optimum pH of 6.5, and it showed activity to pH within the range of 4.5 to 8.0 (Aanangi et al., 2016). In the present study, the LP_PP_SB-treated group exhibited the lowest pH (4.26), which could inhibit lipoxygenase activity more effectively than other treated groups and the control group (pH 4.44–5.30), resulting in the production of more PUFA content, which is consistent with the findings of a previous study (Liu et al., 2023). However, the results of a previous study were contrary to our results; inoculants of *Lactobacillus buchneri* or *L. plantarum* decrease the content of PUFAs as LABs have the ability to promote the biohydrogenation of fatty acids (Liu et al., 2018). It is evident that the combination of *L. plantarum, P. pentosaceus*, and *S. bovis* in the LP_PP_SB-treated group did not promote the biohydrogenation of fatty acids, which may be due to strain specificity in terms of biohydrogenation, and the mechanisms need to be further studied.

### 4.5 Bacterial community of mulberry ensiled for 60 days

*Firmicute*s and *Proteobacteria* were the dominant phyla in both the control and SB-treated groups. This result was consistent with the results of a previous study (Wang et al., 2022). However, the relative abundance of *Proteobacteria* was reduced in other treatment groups. *Firmicutes* and *Proteobacteria* were the most common phyla in silage, owing to their ability to adapt to anaerobic and acidic environments (Wang et al., 2022). pH (5.30) of the control group was the highest, and previous studies had indicated that an increased pH promotes the growth of *Proteobacteria* compared to *Firmicutes* (Tian et al., 2021).

As shown in Figure 3C, the most dominant bacteria at the genus level after 60 days of ensiling were *Lactiplantibacillus, Pediococcus*, and *Enterobacter*. *Lactiplantibacillus* was predominant in the LP and LP_SB inoculated groups, with relative abundances of 82.87% and 81.84%, respectively. *Pediococcus* was predominant in the PP-, PP_SB-, and LP_PP_SB-inoculated groups (relative abundances of these bacteria were 74.67%, 85.27%, and 74.27%, respectively). These results suggest that PP combined with SB showed a higher capacity to facilitate *Pediococcus* activity. Furthermore, the pH in the PP-, PP_SB-, and LP_PP_SB-treated groups was 4.58, 4.70, and 4.26, respectively. A previous study observed that *Pediococcus* can grow well under a pH range of 4.3–4.9 during ensiling (Wang et al., 2022). Additionally, the inoculation of *Pediococcus* was 1 × 10^6^ log_10_ CFU/g FW, 5 × 10^5^ log_10_ CFU/g FW, and 5 × 10^5^ log_10_ CFU/g FW in these groups, respectively. Theoretically, in an environment with an adequate pH and inoculation, the relative abundance of *Pediococcus* should likely be higher in the PP-treated group but lower in the PP_SB- and LP_PP_SB-treated groups. Thus, the interactions between these inoculants may be the main factors responsible for this difference. The results of the present study indicate antagonistic effects between *Lactiplantibacillus* and *Pediococcus* but synergistic effects between *Streptococcus* and *Pediococcus* during ensiling. Previously, we observed antagonistic effects between *Lactiplantibacillus* and *Pediococcus* due to these two bacteria responding in opposite ways to the regulation of metabolites during ensiling (Huang et al., 2022). The results of the present study suggested that inoculation with *L. plantarum* facilitates *Lactiplantibacillus* growth when inoculated alone or with other LABs during ensiling. Notably, inoculation with *P. pentosaceus* and *S. bovi*s showed a greater capacity to facilitate *Pediococcus* activity, even when combined with *L. plantarum, P. pentosaceus*, and *S. bovi*s, indicating that *S. bovi*s was likely to have the capacity to promote *Pediococcus* activity during ensiling.

*Enterobacter* was predominant in the control and the SB-treated groups (with relative abundances of 41.18% and 32.25%, respectively) but was less abundant in other groups. *Enterobacter* can compete with LABs for fermentation substrates during silage, converting fermentation substrates into AN by decarboxylation and deamination of amino acids, resulting in an increased buffering capacity and counteracting the rapid lowering of pH (Pahlow et al., 2015), eventually leading to failed silage fermentation. In this study, the pH values in the groups inoculated with SB, PP, LP_SB, and PP_SB were all >4.5 and showed no differences among the inoculation groups (*P* > 0.05). *Enterobacter* activity is inhibited at pH < 4.5 (Pahlow et al., 2015). Theoretically, *Enterobacter* activity should be the same in this environment for each inoculation group. However, in the present study, the relative abundance of *Enterobacter* in the present study was dominant only in SB among the inoculation groups. This finding suggests that the inoculation of SB with other LABs can decrease *Enterobacter* activity during ensiling. However, the mechanisms underlying this effect require further study. Furthermore, the pH of the LP_PP_SB-treated group was the lowest (pH 4.26) among the control and other inoculation groups (*P* < 0.05), indicating that LABs could inhibit *Enterobacter* activity by decreasing the pH to below 4.5. Overall, the dominant *Enterobacter* in the SB-treated groups suggested that *S. bovis* could be an efficient LAB inoculum for mulberry silage only when combined with other LABs, particularly in combination with *P. pentosaceus* and *L. plantarum*.

### 4.6 Correlation between the characteristics and bacterial community of mulberry silage

*Pediococcus* was significantly positively correlated with LA (R = 0.37, *P* < 0.05), but *Enterobacter* was significantly negatively correlated with LA (R = −0.45, *P* < 0.05). LA has the lowest p*K*a compared with other organic acids, resulting in more rapid dissociation (Partanen, 2000). Thus, LA accumulation in silage was the main reason for the decrease in pH. Usually, well-preserved silage has a high LA content (Weinberg et al., 1993). The inhibition of *Enterobacter* activity at pH values below 4.5 (Pahlow et al., 2015) indicates a negative correlation with LA in the silage system.

*Lactiplantibacillus* was significantly positively correlated with AA (R = 0.46, *P* < 0.01), whereas *Pediococcus* was significantly negatively correlated with AA. *Pediococcus* was a homofermentative LAB (Oliveira et al., 2017). It has been reported that *Lactiplantibacillus* strains were heterofermentative (Lin et al., 2022). In general, homofermentative LABs strictly produce LA, whereas heterofermentative LABs produce both LA and AA during ensiling. The results of the present study revealed that the AA content was lower in the PP_SB- and LP_PP_SB-inoculated groups. The relative abundance of *Pediococcus* in the two inoculation groups was higher than those in the other treatment groups (*P* < 0.01). Thus, these results suggest that the other inoculation groups (except for PP_SB and LP_PP_SB) contained some level of heterofermentative LAB from *Lactiplantibacillus*. *Enterobacter* (R = 0.58, *P* < 0.001) was positively correlated with AN. *Enterobacter* was inhibited when pH < 4.5 (Huang et al., 2022) and can produce AN via the decarboxylation and deamination of amino acids (Pahlow et al., 2015). In this study, *Enterobacter* was found to have a higher relative abundance in the control and the SB-treated groups, which was associated with higher pH values. The results showed that the CP content was positively correlated with the relative abundance of *Lactobacillus*. Proteins are degraded by plant proteases and microbial enzymes (Xian et al., 2022), and LABs convert WSC into LA, promoting rapid acidification to inhibit proteolysis (Ali et al., 2020). In this study, the DM content was found to be positively correlated with *Bifidobacterium*, which, along with facultative anaerobes, are harmful to silage preservation (Zheng et al., 2017). However, the role of *Bifidobacterium* remains unclear and will require further investigation.

### 4.7 Metabolite profile of mulberry silage

Metabolites of mulberry silage-inoculated LAB strains were at a distance from the control group (Figure 5), suggesting that inoculated *L. plantarum* or *P. pentosaceus* influenced metabolite compositions of mulberry silage by the microbial community. This finding was consistent with the findings of a previous study (Guo et al., 2022). LAB could convert and degrade phenolic compounds by various enzymes such as tannase, β-glucosidase, and phenolic acid decarboxylase (De Montijo-Prieto et al., 2023). The results of the present study showed that apigenin was upregulated in the PP-treated group, kaempferol-3-o-glucoside and rutin were upregulated in the LP_PP- and LP_PP_SB-treated groups, and quercetin-3-glucoside was upregulated in the LP-, LP_PP-, and LP_PP_SB-treated groups (Figure 8B). The upregulation of these five flavonoids in different treated groups may be attributed to LAB strain specificity in enzymatic types and enzymatic activity aspects. Eriodictyol had the highest abundance in the LP_SB- and LP_PP_SB-treated groups with the highest level of antioxidant capacity, was moreover upregulated in each treated group (except for the SB-treated group), and showed a significant positive correlation with ABTS and FRAP (Figure 8C). Therefore, eriodictyol was the most important than other four flavonoids for antioxidant capacity of mulberry silage. Eriodictyol had the highest abundance in the LP_SB- and LP_PP_SB-treated groups, indicating that the combination of *L. plantarum* with *P. pentosaceus* was conducive to the conversion of other phenolic compounds to eriodictyol. Numerous studies have demonstrated the remarkable antioxidant potential of eriodictyol. A previous study observed that eriodictyol derived from *Afzelia africana* exhibits strong antioxidant properties against ABTS and DDPH radicals of 2.1 and 2.5 μg/ml, respectively (Vigbedor et al., 2023). Mice received eriodictyol by intraperitoneal injections at doses of 10, 20, and 40 mg/kg for 3 consecutive days, and it was observed that the production of glutathione (GSH), superoxide dismutase (SOD), and CAT significantly upregulated and inhibited the production of malondialdehyde (MDA), thiobarbituric acid (TBARS), and ROS in eriodictyol-treated group compared with the control group (Li et al., 2016). Similarly, eriodictyol of 50 or 200 mg/kg alleviated oxidative stress of hepatotoxicity in mice induced by acetaminophen due to the increased content of GSH and activity of SOD and decreased content of MDA (Ye et al., 2017.). In the present study, eriodictyol was upregulated in the inoculated groups. A mixture of flax seed extract and soy was separately inoculated into 25 strains of *Bifidobacterium* and subjected to anaerobic fermentation at 37°C for 5 days, and it was observed that eriodictyol content increased among fermented extracts of 24 *Bifidobacterium* strains in the treated groups (0.34–0.7 mg/L of fermentation media) compared to the control group (0.29 mg/L of fermentation media) (Peirotén et al., 2020). In addition, a previous study also showed that eriodictyol was continuously increased (~0%−100%) in the water extract of *Fructus aurantii immaturus* incubated with human intestinal bacteria under anaerobic conditions for 0, 12, 24, and 48 h at 37 °C (Liu et al., 2017). This result may suggest that LAB increases the antioxidant capacity of fermentation by converting other compounds into eriodictyol, which has more antioxidant capacity. Eriodictyol belongs to isoflavone aglycones (Peirotén et al., 2020), and it has been reported that intestinal microbes can convert plant lignans and flavonoids into aglycones. These aglycones exhibit higher antioxidant capacity than their respective glycosides (lignans and flavonoids) (Landete et al., 2016). In the present study, the ensiling of mulberry was carried out by inoculation with lactic acid bacteria fermented for a longer period of time (60 days) than in the two studies mentioned above (5 days and 48 h). This result probably indicated that eriodictyol could still be stabilized even after a long time of fermentation. However, the present study was unable to quantify the contribution of lactic acid bacteria with antioxidant properties in complex fermentation systems to the antioxidant capacity of silage, which requires further exploration in future research.

## 5 Conclusion

The LAB strains isolated from naturally fermented mulberry silage, *L. plantarum* and *P. pentosaceus*, can both increase DM content and decrease the pH of mulberry silage to different extents, whether inoculated in combination or alone. Particularly, the combination (the LP_PP_SB-treated group) of *L. plantarum, P. pentosaceus*, and *S. bovis* could significantly increase the abundance of *Lactiplantibacillus* and *Pediococcus* and decrease the relative abundance of *Enterobacter*, increase the concentration of LA, decrease the pH and concentration of AN, and increase the DM content so as to improve the quality of mulberry silage. However, an inoculant of *S. bovis* isolated from cattle could improve mulberry silage and reduce the concentration of AN only when combined with *L. plantarum* or *P. pentosaceus*. In addition, the LAB strains enhanced the DPPH, ABTS, and FRAP levels of mulberry silage, thereby increasing antioxidant capacity, with the highest levels observed in the LP_PP- and LP_PP_SB-treated groups. Untargeted metabolomics analysis revealed that five flavonoids (apigenin, eriodictyol, quercetin-3-glucoside, rutin, and kaempferol-3-O-rutinoside) associated with antioxidant capacity were upregulated upon inoculation (except for SB-treated groups), among which the stability of eriodictyol might be the key factor in the enhancement of antioxidant capacity of the silage. Overall, the combination (the LP_PP_SB-treated group) of *L. plantarum, P. pentosaceus*, and *S. bovis* could improve the fermentation quality and antioxidant capacity of mulberry silage. These findings offer theoretical support for the utilization of LAB in mulberry silage, demonstrating that, by selecting appropriate strains of lactic acid bacteria, both the quality and antioxidant capacity of the silage can be effectively enhanced. This insight may prove valuable for the production and management of mulberry silage.

## Data availability statement

The datasets presented in this study can be found in online repositories. The names of the repository/repositories and accession number(s) can be found at: PRJNA949551.

## Author contributions

YG: Conceptualization, Data curation, Investigation, Visualization, Writing – original draft, Writing – review & editing. RH: Conceptualization, Writing – review & editing. YN: Formal analysis, Methodology, Writing – review & editing. PZ: Investigation, Methodology, Writing – review & editing. YL: Formal analysis, Investigation, Writing – review & editing. WZ: Conceptualization, Writing – review & editing.
