# Supplementary material for: Chemical characteristics, antioxidant capacity, bacterial community, and metabolite composition of mulberry silage ensiling with lactic acid bacteria
Source: Front Microbiol. 2024 Apr 8;15:1363256. doi: 10.3389/fmicb.2024.1363256 (PMC11033325; doi:10.3389/fmicb.2024.1363256)
Supplement: Supplementary file 1 [file Table_1.DOCX]

| Items | LM14 | LM15 | LM17 | PP18 | LP19 | LM20 | LM21 | LM22 | LM23 | PP25 | LP26 | LM27 |
| --- | --- | --- | --- | --- | --- | --- | --- | --- | --- | --- | --- | --- |
| OD (600nm) | 2.085 | 2.136 | 2.14 | 2.139 | 2.157 | 2.081 | 2.158 | 1.908 | 1.878 | 2.079 | 2.155 | 1.832 |
| pH | 3.78 | 3.79 | 3.79 | 3.89 | 3.78 | 3.91 | 3.79 | 4.16 | 4.31 | 3.89 | 3.79 | 4.37 |

**Table S1.** OD（600nm） and pH value of 12 LAB strains isolated from natural fermented mulberry
