# Supplementary material for: Chemical characteristics, antioxidant capacity, bacterial community, and metabolite composition of mulberry silage ensiling with lactic acid bacteria
Source: Front Microbiol. 2024 Apr 8;15:1363256. doi: 10.3389/fmicb.2024.1363256 (PMC11033325; doi:10.3389/fmicb.2024.1363256)
Supplement: Supplementary file 2 [file Table_2.DOCX]

**Table S2** Fifty-nine different metabolites involved in the biosynthesis of other secondary metabolites pathway.

| serial number | Metabolite | Formula | Retention time | Mode | HMDB Superclass | HMDB Class | HMDB Subclass | M/Z |
| --- | --- | --- | --- | --- | --- | --- | --- | --- |
| 1 | Rutin | C_27_H_30_O_16_ | 4.97 | pos | Phenylpropanoids and polyketides | Flavonoids | Flavonoid glycosides | 611.16 |
| 2 | L-(+)-Arginine | C_6_H_14_N4O_2_ | 0.76 | pos | Organic acids and derivatives | Carboxylic acids and derivatives | Amino acids, peptides, and analogues | 175.12 |
| 3 | L-Isoleucine | C_6_H_13_NO_2_ | 2.24 | pos | Organic acids and derivatives | Carboxylic acids and derivatives | Amino acids, peptides, and analogues | 132.10 |
| 4 | Kaempferol-3-O-rutinoside | C_27_H_30_O_15_ | 5.18 | pos | Phenylpropanoids and polyketides | Flavonoids | Flavonoid glycosides | 595.16 |
| 5 | 5-Aminopentanal | C_5_H_11_NO | 0.05 | pos | Organic oxygen compounds | Organooxygen compounds | Carbonyl compounds | 102.09 |
| 6 | DL-Pipecolinic acid | C_6_H_11_NO_2_ | 0.68 | pos | Organic acids and derivatives | Carboxylic acids and derivatives | Amino acids, peptides, and analogues | 147.11 |
| 7 | Trans-o-Coumaric acid 2-glucoside | C_15_H_18_O_8_ | 5.10 | pos | Organic oxygen compounds | Organooxygen compounds | Carbohydrates and carbohydrate conjugates | 390.12 |
| 8 | Glyceollin III | C_20_H_18_O_5_ | 5.27 | pos | Phenylpropanoids and polyketides | Isoflavonoids | Furanoisoflavonoids | 371.15 |
| 9 | Pinobanksin 3-O-acetate | C_17_H_14_O_6_ | 5.76 | pos | - | - | - | 315.09 |
| 10 | Quercetin | C_15_H_10_O_7_ | 5.98 | pos | Phenylpropanoids and polyketides | Flavonoids | Flavones | 303.05 |
| 11 | Quercetin 3-methyl ether | C_16_H_12_O_7_ | 6.10 | pos | - | - | - | 317.06 |
| 12 | Apigenin | C_15_H_10_O_5_ | 6.42 | pos | Phenylpropanoids and polyketides | Flavonoids | Flavones | 271.06 |
| 13 | Farnesyl pyrophosphate | C_15_H_28_O_7_P_2_ | 7.02 | pos | Lipids and lipid-like molecules | Prenol lipids | Sesquiterpenoids | 421.09 |
| 14 | Chrysin | C_15_H_10_O_4_ | 7.16 | pos | Phenylpropanoids and polyketides | Flavonoids | Flavones | 255.06 |
| 15 | UMBELLIFERONE | C_9_H_6_O_3_ | 14.84 | pos | Phenylpropanoids and polyketides | Coumarins and derivatives | Hydroxycoumarins | 163.04 |
| 16 | Cephalosporin C | C_16_H_21_N_3_O_8_S | 7.05 | pos | Organic acids and derivatives | Carboxylic acids and derivatives | Amino acids, peptides, and analogues | 416.11 |
| 17 | 5-Hydroxy-1-(4-hydroxyphenyl)-3-decanone | C_16_H_24_O_3_ | 7.02 | pos | - | - | - | 265.18 |
| 18 | (-)-8-Demethylmaritidine | C_16_H_19_NO_3_ | 6.99 | pos | - | - | - | 274.14 |
| 19 | (7R)-7-(5-Carboxy-5-oxopentanoyl)aminocephalosporinate | C_16_H_18_N_2_O_9_S | 6.68 | pos | Organoheterocyclic compounds | Lactams | Beta lactams | 432.11 |
| 20 | N1,N5,N10-Tricoumaroyl spermidine | C_34_H_37_N_3_O_6_ | 5.93 | pos | - | - | - | 584.27 |
| 21 | Estragole | C_10_H_12_O | 5.66 | pos | Benzenoids | Phenol ethers | Anisoles | 149.10 |
| 22 | Matairesinol | C_20_H_22_O_6_ | 5.42 | pos | Lignans, neolignans and related compounds | Furanoid lignans | Tetrahydrofuran lignans | 359.15 |
| 23 | 6''-Acetylapiin | C_28_H_30_O_15_ | 5.30 | pos | Benzenoids | Naphthalenes | Phenylnaphthalenes | 571.14 |
| 24 | Sinapyl alcohol | C_11_H_14_O_4_ | 5.17 | pos | Benzenoids | Phenols | Methoxyphenols | 211.10 |
| 25 | 6,7-Dihydroxycoumarin | C_9_H_6_O_4_ | 4.86 | pos | Phenylpropanoids and polyketides | Coumarins and derivatives | Hydroxycoumarins | 179.03 |
| 26 | Biochanin A 7-(6-malonylglucoside) | C_25_H_24_O_13_ | 4.80 | pos | Phenylpropanoids and polyketides | Isoflavonoids | Isoflavonoid O-glycosides | 533.13 |
| 27 | L-Tryptophan | C_11_H_12_N_2_O_2_ | 4.61 | pos | Organoheterocyclic compounds | Indoles and derivatives | Indolyl carboxylic acids and derivatives | 409.19 |
| 28 | L-Proline | C_5_H_9_NO_2_ | 4.55 | pos | Organic acids and derivatives | Carboxylic acids and derivatives | Amino acids, peptides, and analogues | 116.07 |
| 29 | Acetyltropine | C_10_H_17_NO_2_ | 3.14 | pos | - | - | - | 184.13 |
| 30 | (S)-2-amino-6-oxohexanoate | C_6_H_11_NO_3_ | 1.62 | pos | - | - | - | 187.11 |
| 31 | Ecgonine | C_9_H_15_NO_3_ | 1.59 | pos | Alkaloids and derivatives | Tropane alkaloids | Not Available | 218.14 |
| 32 | Swainsonine | C_8_H_15_NO_3_ | 0.82 | pos | - | - | - | 174.11 |
| 33 | Spermidine | C_7_H_19_N_3_ | 0.70 | pos | Organic nitrogen compounds | Organonitrogen compounds | Amines | 146.16 |
| 34 | Caffeic acid | C_9_H_8_O_4_ | 6.14 | pos | Phenylpropanoids and polyketides | Cinnamic acids and derivatives | Hydroxycinnamic acids and derivatives | 163.04 |
| 35 | Trans-Cinnamic acid | C_9_H_8_O_2_ | 4.55 | pos | Phenylpropanoids and polyketides | Cinnamic acids and derivatives | Cinnamic acids | 166.09 |
| 36 | Kaempferol-3-O-glucoside | C_21_H_20_O_11_ | 5.03 | neg | Phenylpropanoids and polyketides | Flavonoids | Flavonoid glycosides | 447.09 |
| 37 | Gallic acid | C_7_H_6_O_5_ | 5.55 | neg | Benzenoids | Benzene and substituted derivatives | Benzoic acids and derivatives | 151.00 |
| 38 | Xanthine | C_5_H_4_N_4_O_2_ | 0.90 | neg | Organoheterocyclic compounds | Imidazopyrimidines | Purines and purine derivatives | 151.03 |
| 39 | (-)-Shikimic acid | C_7_H_10_O_5_ | 3.08 | neg | Organic oxygen compounds | Organooxygen compounds | Alcohols and polyols | 173.05 |
| 40 | Vanillin | C_8_H_8_O_3_ | 4.15 | neg | Benzenoids | Phenols | Methoxyphenols | 151.04 |
| 41 | Chlorogenic Acid | C_16_H_18_O_9_ | 4.20 | neg | Organic oxygen compounds | Organooxygen compounds | Alcohols and polyols | 353.09 |
| 42 | 5-Hydroxyferulate acid | C_10_H_10_O_5_ | 4.72 | neg | Phenylpropanoids and polyketides | Cinnamic acids and derivatives | Hydroxycinnamic acids and derivatives | 209.05 |
| 43 | Trans-Resveratrol | C_14_H_12_O_3_ | 5.36 | neg | Phenylpropanoids and polyketides | Stilbenes | Not Available | 227.07 |
| 44 | Scolymoside | C_27_H_30_O_14_ | 5.59 | neg | Phenylpropanoids and polyketides | Tannins | Hydrolyzable tannins | 615.12 |
| 45 | Eriodictyol | C_15_H_12_O_6_ | 5.68 | neg | Phenylpropanoids and polyketides | Flavonoids | Flavans | 287.06 |
| 46 | Luteolin | C_15_H_10_O_6_ | 6.23 | neg | Phenylpropanoids and polyketides | Flavonoids | Flavones | 571.09 |
| 47 | Glycitin | C_22_H_22_O_10_ | 6.38 | neg | Phenylpropanoids and polyketides | Isoflavonoids | Isoflavonoid O-glycosides | 467.10 |
| 48 | Betanin | C_24_H_26_N_2_O_13_ | 5.86 | neg | Lipids and lipid-like molecules | Prenol lipids | Sesquiterpenoids | 585.11 |
| 49 | Quercetin-3-glucoside | C_21_H_20_O_12_ | 4.71 | neg | Phenylpropanoids and polyketides | Flavonoids | Flavonoid glycosides | 463.09 |
| 50 | 4-Hydroxyphenylacetaldehyde | C_8_H_8_O_2_ | 4.47 | neg | Benzenoids | Benzene and substituted derivatives | Phenylacetaldehydes | 135.05 |
| 51 | Myricetin | C_15_H_10_O_8_ | 4.41 | neg | Phenylpropanoids and polyketides | Flavonoids | Flavones | 317.03 |
| 52 | 3,4-Dihydroxyphenylacetaldehyde | C_8_H_8_O_3_ | 3.93 | neg | Benzenoids | Benzene and substituted derivatives | Phenylacetaldehydes | 197.05 |
| 53 | Cucurbitacin B | C_32_H_46_O_8_ | 3.92 | neg | Lipids and lipid-like molecules | Steroids and steroid derivatives | Cucurbitacins | 595.26 |
| 54 | L-Tyrosine | C_9_H_11_NO_3_ | 2.69 | neg | Organic acids and derivatives | Carboxylic acids and derivatives | Amino acids, peptides, and analogues | 180.07 |
| 55 | L-Glutamic acid | C_5_H_9_NO_4_ | 1.76 | neg | Organic acids and derivatives | Carboxylic acids and derivatives | Amino acids, peptides, and analogues | 128.04 |
| 56 | 1-Pyrroline-5-carboxylic acid | C_5_H_7_NO_2_ | 3.91 | neg | Organic acids and derivatives | Carboxylic acids and derivatives | Amino acids, peptides, and analogues | 285.11 |
| 57 | Epicatechin | C_15_H_14_O_6_ | 5.24 | neg | Phenylpropanoids and polyketides | Flavonoids | Flavans | 289.07 |
| 58 | Kanamycin | C_18_H_36_N_4_O_11_ | 6.33 | neg | Organic oxygen compounds | Organooxygen compounds | Carbohydrates and carbohydrate conjugates | 521.18 |
| 59 | Neomycin | C_23_H_46_N_6_O_13_ | 8.96 | neg | Organic oxygen compounds | Organooxygen compounds | Carbohydrates and carbohydrate conjugates | 595.29 |
