# Supplementary material for: Chemical characteristics, antioxidant capacity, bacterial community, and metabolite composition of mulberry silage ensiling with lactic acid bacteria
Source: Front Microbiol. 2024 Apr 8;15:1363256. doi: 10.3389/fmicb.2024.1363256 (PMC11033325; doi:10.3389/fmicb.2024.1363256)
Supplement: Supplementary file 3 [file Table_3.DOCX]

**Table S3.** Relative abundance of upregulated five flavonoids in PP, LP, LP_PP, LP_SB, PP_SB and LP_PP_SB treated groups and control, respectively.

| **Groups** | **Apigenin** | **Eriodictyol** | **kaempferol-3-o-glucoside** | **quercetin-3-glucoside** | **rutin** |
| --- | --- | --- | --- | --- | --- |
| CON | 2.33 | 3.15 | 4.09 | 3.96 | 4.22 |
| PP | 3.47* | 3.41*** | no significant difference | no significant difference | no significant difference |
| LP | no significant difference | 3.53*** | 5.1791*** | 5.57*** | 4.98* |
| LP_PP | no significant difference | 3.65*** | no significant difference | 4.88** | no significant difference |
| LP_SB | no significant difference | 3.67*** | no significant difference | no significant difference | no significant difference |
| PP_SB | no significant difference | 3.44* | no significant difference | no significant difference | no significant difference |
| LP_PP_SB | no significant difference | 3.62*** | 5.13*** | 5.51*** | 4.86* |

* Represent statistically significant differences between control and each treated group, *, *P* < 0.05, **, *P* < 0.01, ***, *P* < 0.001.
