# Supplementary material for: Chemical characteristics, antioxidant capacity, bacterial community, and metabolite composition of mulberry silage ensiling with lactic acid bacteria
Source: Front Microbiol. 2024 Apr 8;15:1363256. doi: 10.3389/fmicb.2024.1363256 (PMC11033325; doi:10.3389/fmicb.2024.1363256)
Supplement: Supplementary file 4 [file Table_4.DOCX]

Supplementary Figures title

**Figure S1.** Phylogenetic tree of 12 LAB strains derived from 16S rDNA sequence analysis.

**Figure S2.** Permutation testing of PLS_DA model.

**Figure S3.** Different metabolites between control and each treated group in Biosynthesis of secondary metabolites pathway.
