# Supplementary figures and images for: Chemical characteristics, antioxidant capacity, bacterial community, and metabolite composition of mulberry silage ensiling with lactic acid bacteria
Source: Front Microbiol. 2024 Apr 8;15:1363256. doi: 10.3389/fmicb.2024.1363256 (PMC11033325; doi:10.3389/fmicb.2024.1363256)

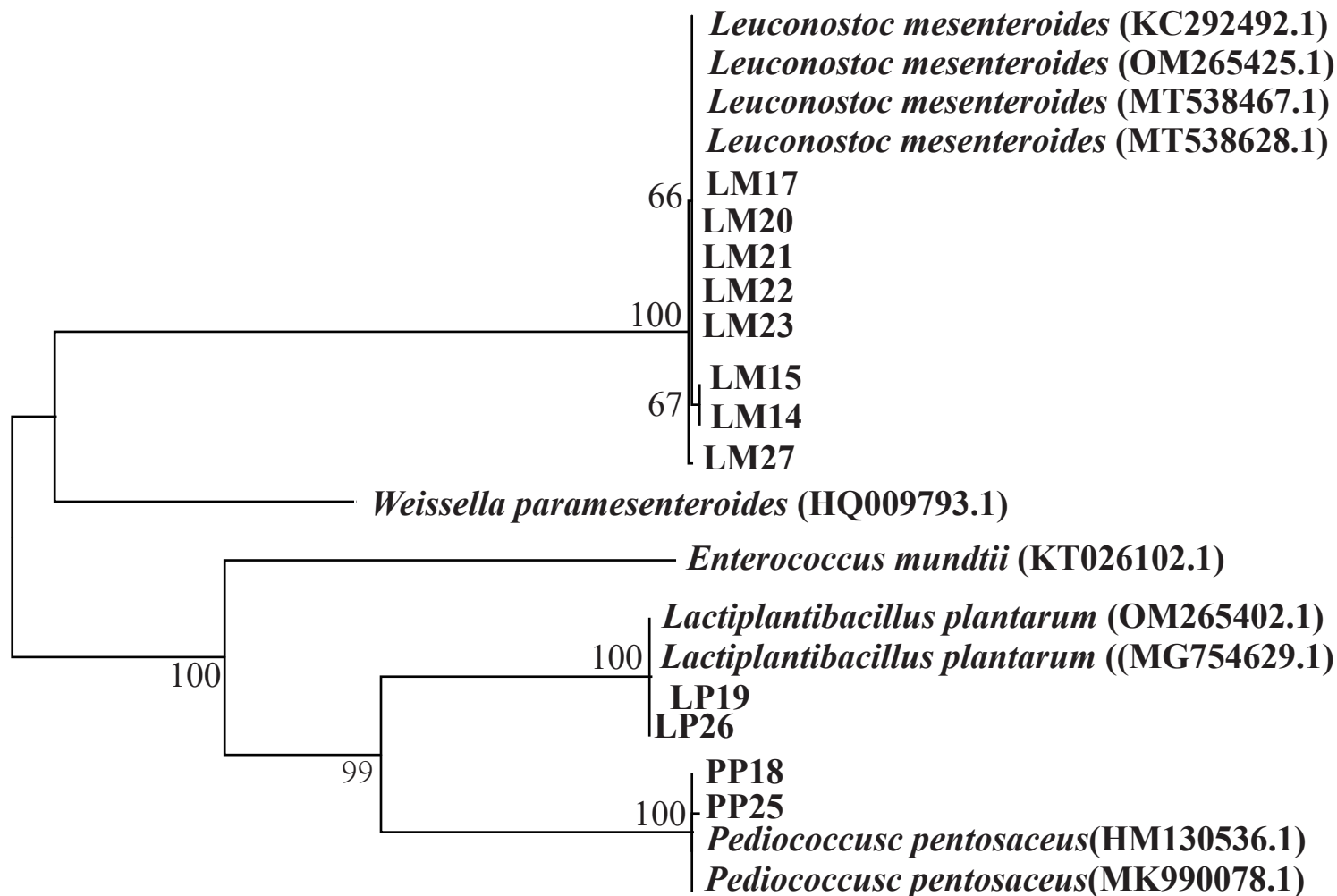

0.01

Supplement: Supplementary file 5 [file Image_1.pdf]

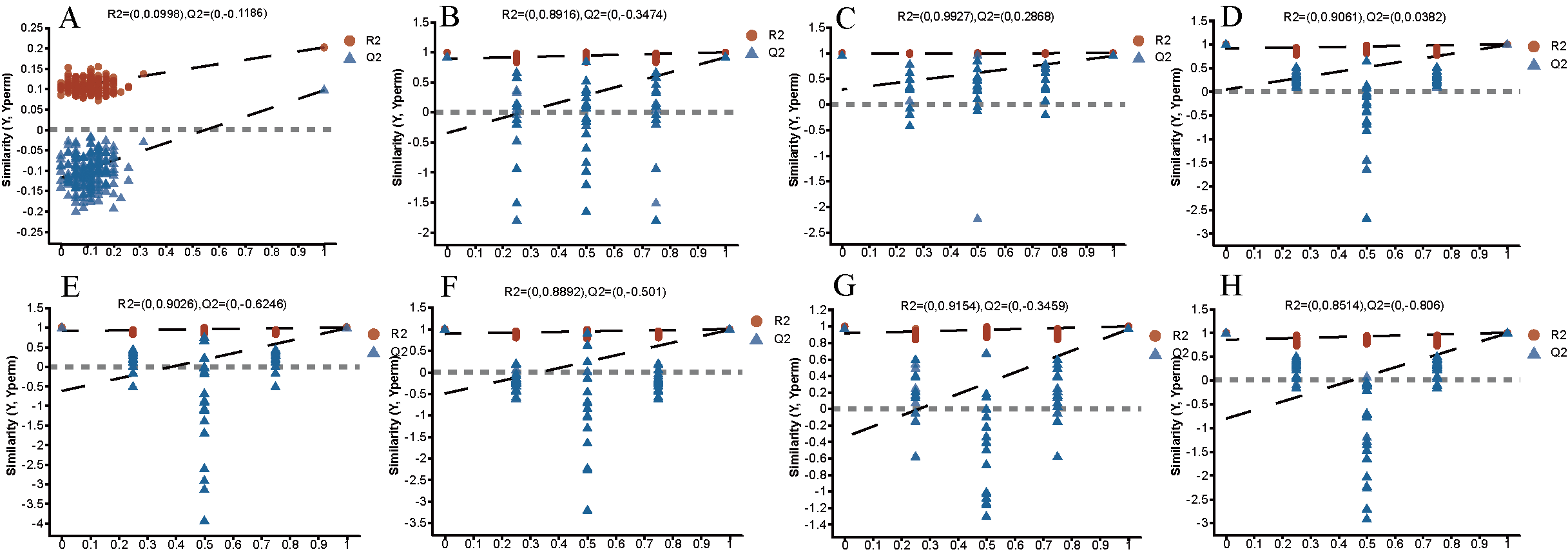

Supplement: Supplementary file 6 [file Image_2.TIF]

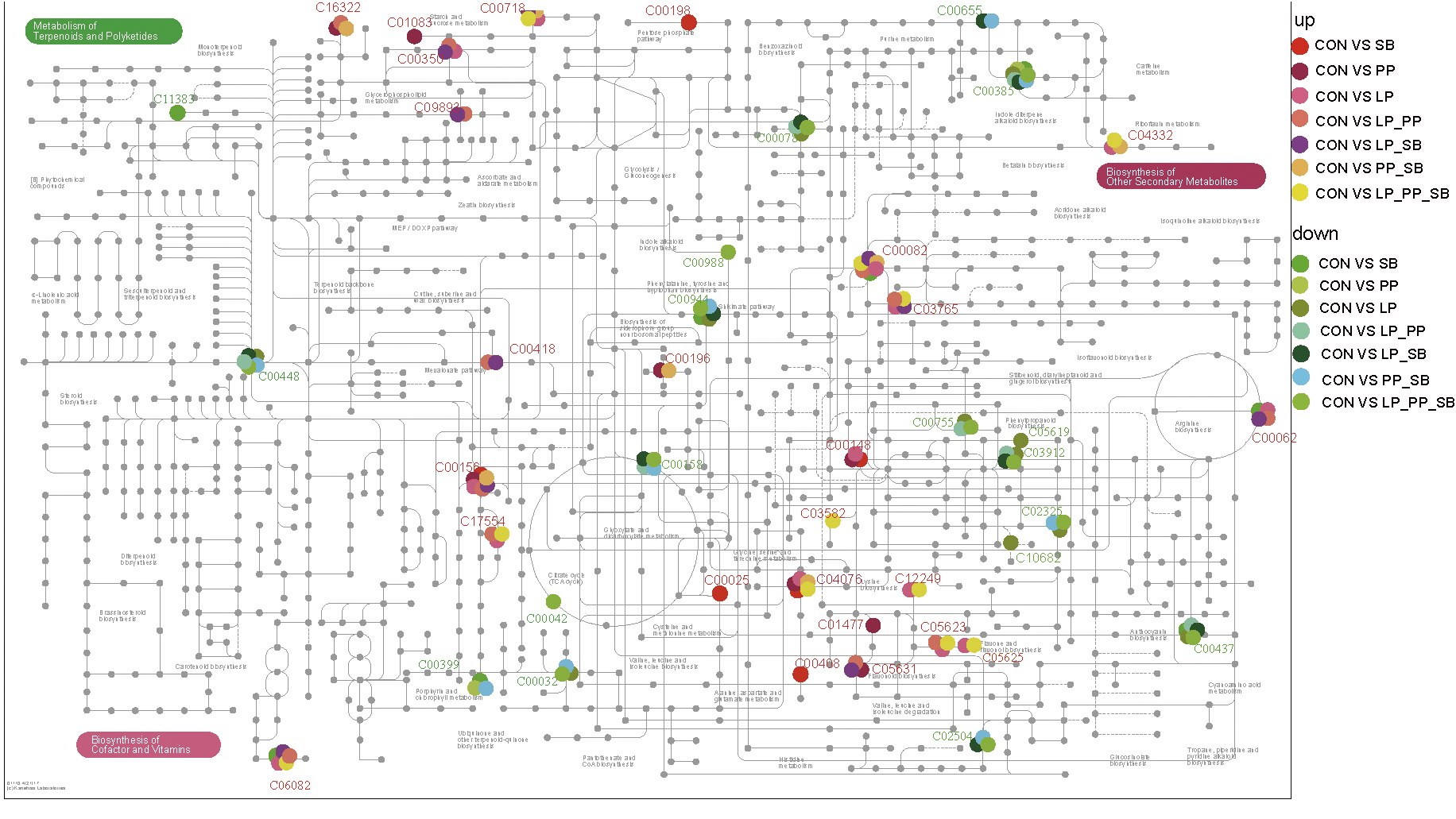

Supplement: Supplementary file 7 [file Image_3.TIF]
